# Supplementary material for: Stable mid-infrared polarization imaging based on quasi-2D tellurium at room temperature
Source: Nat Commun. 2020 May 8;11:2308. doi: 10.1038/s41467-020-16125-8 (PMC7210936; doi:10.1038/s41467-020-16125-8)
Supplement: Supplementary file 1 — Supplementary Information [file 41467_2020_16125_MOESM1_ESM.pdf]

Supplementary Information for
Stable mid-infrared polarization imaging based on quasi-2D tellurium at room temperature

Tong et al.

Supplementary Note 1. Crystal characterization

The optical image of tellurium nanoflake is displayed in Supplementary Figure 1a, and its Raman spectra results are shown in Supplementary Figure 1b, revealing the corresponding characteristic peaks which are located at 96.5 cm^{-1}, 124.3 cm^{-1}, and 144.6 cm^{-1}, respectively corresponding to three Raman active modes of E_1-TO, A_1 and E_2. The narrow full width at half maximum (FWHM) of A_1 peak suggests the high crystal quality. The Raman mapping results of E_1-TO, A_1 and E_2 mode intensity and peak location, are shown in Supplementary Figure 1c-1h, respectively, the uniform intensity and peak location indicate high quality of our sample. Atomic force microscope (AFM) is also measured for the sample in Supplementary Figure 1a, as shown in Supplementary Figure 1i-1j. Supplementary Figure 1i and 1j show the height distribution along two perpendicular directions marked by white and red dashed lines in Supplementary Figure 1i, the thickness of Te is ~27.5 nm. We have chosen thick sample because the absorption edge can be redshifted for longer wavelength, which is beneficial for MIR photodetecting and imaging, and the absorption can be stronger for thick samples, transmitted light into the substrate can also be limited. To verify anisotropic electronic characters of Te, we have fabricated another device with two perpendicular pairs of electrodes, the channel lengths are 10 μm for both directions to ensure precise comparison in Figure 1e-1g, the optical image of this device is shown in Supplementary Figure 1k.

Supplementary Note 2. Drain current analysis for different channel length

Based on transfer curves in Figure 1d, here we focus on the curve for the longest channel length of 16 μm, the drain bias is fixed at 1.0 V, the device shows an on-off ratio of 1.48 × 10^3, and the tellurium nanoflake is an intrinsic p-type semiconductor. From the transfer curve, the calculated field effect mobility is μ=~900 $cm^2V^{-1}s^{-1}$ (details included in Supplementary Note 3), which is much higher comparing with other transition metal dichalcogenides (Supplementary Table 1, Supplementary Figure 26). For the longest channel length, drain current under different gate bias is also measured in Supplementary Figure 2a and 2b, which is in accordance with transfer curves in Figure 1d. The maximum on current under gate bias of 10.0 V is summarized in Supplementary Figure 2c, which is decreasing with longer channel length based on transfer curves in Figure 1d.

The drain currents are measured under drain bias from -1.0 V to 1.0 V, for different channel lengths at room temperature, as shown in Supplementary Figure 3a, the gate bias is 0 V, the relationship between drain current and channel length is also studied under 1.0 V drain bias, to indicate larger drain current generated by narrower channel length, as shown in the inset of Supplementary Figure 3a. This is mainly attributed to two reasons, one is the channel resistance R_{ch} which is proportional to the channel length, and the other is the carrier velocity which is increasing with the decrease of the channel length.

Based on transfer curves in Figure 1d, in Supplementary Figure 3b, the on-off ratio is slightly decreased in shorter channel length, which is due to weaker gate control over the drain current.

The characteristic length for short channel effect is defined by Supplementary Equation 1:

$$\lambda_{sc}=\sqrt{\frac{\varepsilon_{Te}}{\varepsilon_{SiO_2}}d_{Te}d_{SiO_2}}, \qquad (1)$$

where ε_{Te}=28.0 and ε_{SiO_2}=3.9 are the relative permittivity of tellurium and SiO_2, d_{Te} and d_{SiO_2} are the thickness of tellurium sample and gate oxide (SiO_2). The calculated λ_{sc}=236.6 nm, so short channel effect is excluded for our sample.

In Supplementary Figure 3c, we have calculated the field effect carrier mobility for different channel length. With longer channel length L_{ch}, the transconductance is decreasing rapidly, as the transconductance is linearly proportional to $\frac{1}{L_{ch}}$. For the shortest channel length L_{ch}=1 μm, the transconductance is g_m=25.95 μS, for the longest channel length L_{ch}=16 μm, the transconductance is g_m=1.72 μS. As a result, the mobility should be irrelevant with the channel length. In addition, the channel length is large comparing with the characteristic length λ_{sc}, so the carrier velocity is not saturated and the mobility is not limited by the device geometry. Based on these discussions, tellurium can preserve high performance under room temperature and with various device design.

Supplementary Note 3. Field effect mobility analysis

The field effect mobility of our device can be calculated by Supplementary Equation 2 from the transfer curve:

$$\mu=\frac{g_m}{V_{DS}}\times\frac{L_{ch}}{W_{ch}C_{SiO_2}}, \qquad (2)$$

where g_m is the transconductance of tellurium device, L_{ch} and W_{ch} are the channel length and width respectively, and C_{SiO_2} is the capacitance of the gate oxide per unit area. Based on parallel-plate capacitor model, the capacitance of SiO_2 layer is:

$$C_{SiO_2}=\frac{\varepsilon_0\varepsilon_{SiO_2}}{d_{SiO_2}}. \qquad (3)$$

The thickness of SiO_2 is $d_{SiO_2}=300\ \text{nm}$, $\varepsilon_0=8.85\times10^{-12}\text{Fm}^{-1}$, the relative permittivity of SiO_2 is $\varepsilon_{SiO_2}=3.9$, so the SiO_2 capacitance is $C_{SiO_2}=115.1\ \mu\text{Fm}^{-2}$. By differentiate the transfer curve in Figure 1d, we can calculate the transconductance to be $g_m=1.72\ \mu\text{S}$. Then the calculated field effect mobility is $\mu=938\ \text{cm}^2\text{V}^{-1}\text{s}^{-1}$, which is much higher comparing with other transition metal dichalcogenides (Supplementary Table 1).

Supplementary Note 4. Polarized Raman analysis

Quasi-2D tellurium nanoflakes belong to space group D_3^4, the Raman tensor for E_1-TO, A_1 and E_2 active modes can be written as [1,2]:

$$\mathbf{R}(E_1\text{-}TO)=\begin{bmatrix} a & 0 & 0 \\ 0 & b & c \\ 0 & c & 0 \end{bmatrix}, \quad (4)$$

$$\mathbf{R}(A_1)=\begin{bmatrix} d & 0 & 0 \\ 0 & e & 0 \\ 0 & 0 & f \end{bmatrix}, \quad (5)$$

$$\mathbf{R}(E_2)=\begin{bmatrix} 0 & g & h \\ g & 0 & 0 \\ h & 0 & 0 \end{bmatrix}, \quad (6)$$

where a, b, c, d, e, f, g, h are the Raman tensor elements. In our Raman experiments, the incident laser is linear polarized and illuminated on the sample, the sample is rotated to change the polarization direction. The collected scattering signal is also polarized and the polarization direction is parallel to the incident laser direction. Based on Raman tensor theory, the incident and scattering polarization vector are $e_i=[\sin\theta, 0, \cos\theta]$ and $e_s=e_i=[\sin\theta, 0, \cos\theta]$, where θ is the angle between the longer side of tellurium nanoflake (x axis) and incident polarization direction [3,4]. The Raman intensity can be calculated by Supplementary Equation 7:

$$I= |e_i \cdot \mathbf{R} \cdot e_s|^2. \quad (7)$$

So the polarized Raman intensity for the three modes are:

$$I_{E_1\text{-}TO,\parallel}=|[\sin\theta, 0, \cos\theta]\begin{bmatrix} a & 0 & 0 \\ 0 & b & c \\ 0 & c & 0 \end{bmatrix}\begin{bmatrix} \sin\theta \\ 0 \\ \cos\theta \end{bmatrix}|^2= |a\sin^2\theta|^2, \quad (8)$$

$$I_{A_1,\parallel}= |[\sin\theta, 0, \cos\theta]\begin{bmatrix} d & 0 & 0 \\ 0 & e & 0 \\ 0 & 0 & f \end{bmatrix}\begin{bmatrix} \sin\theta \\ 0 \\ \cos\theta \end{bmatrix}|^2=|d\sin^2\theta+f\cos^2\theta|^2, \quad (9)$$

$$I_{E_2,\parallel}= |[\sin\theta, 0, \cos\theta]\begin{bmatrix} 0 & g & h \\ g & 0 & 0 \\ h & 0 & 0 \end{bmatrix}\begin{bmatrix} \sin\theta \\ 0 \\ \cos\theta \end{bmatrix}|^2=|2h\sin\theta\cos\theta|^2. \quad (10)$$

As a result, E_1-TO and A_1 mode show a two-lobe shape, and E_2 mode show a four-lobe shape.

In addition, the polarized Raman intensity is closely related with the scattering polarization vector, when the scattering signal is polarized perpendicular to the incident laser, the scattering polarization vector changes to be $e_s=[\cos\theta, 0, \sin\theta]$,

then the Raman intensity changes to be:

$$I_{E_1\text{-}TO,\perp}=\left|[\sin\theta,\ 0,\ \cos\theta]\begin{bmatrix} a & 0 & 0 \\ 0 & b & c \\ 0 & c & 0 \end{bmatrix}\begin{bmatrix} \cos\theta \\ 0 \\ \sin\theta \end{bmatrix}\right|^2=|a\sin\theta\cos\theta|^2, \qquad (11)$$

$$I_{A_1,\perp}=\left|[\sin\theta,\ 0,\ \cos\theta]\begin{bmatrix} d & 0 & 0 \\ 0 & e & 0 \\ 0 & 0 & f \end{bmatrix}\begin{bmatrix} \cos\theta \\ 0 \\ \sin\theta \end{bmatrix}\right|^2=|(d+f)\sin\theta\cos\theta|^2, \qquad (12)$$

$$I_{E_2,\perp}=\left|[\sin\theta,\ 0,\ \cos\theta]\begin{bmatrix} 0 & g & h \\ g & 0 & 0 \\ h & 0 & 0 \end{bmatrix}\begin{bmatrix} \cos\theta \\ 0 \\ \sin\theta \end{bmatrix}\right|^2=|h|^2. \qquad (13)$$

Under these conditions, the E_1-TO and A_1 mode will show a four-lobe shape, and E_2 mode is unpolarized.

When no half-wave plate is placed before the detector, all scattering signals are collected, under these conditions, the Raman intensity analysis will be very complicated. To qualitatively fitting the experimental results, we can focus on the scattered signal along two perpendicular directions, where the scattering polarization vector is parallel or perpendicular to the incident polarization vector, based on our previous work about monolayer MoS_2, and the Raman intensity can be written as:

$$I_{E_1\text{-}TO}=I_{E_1\text{-}TO,\parallel}+I_{E_1\text{-}TO,\perp}=|a\sin^2\theta|^2+|a\sin\theta\cos\theta|^2, \qquad (14)$$

$$I_{A_1}=I_{A_1,\parallel}+I_{A_1,\perp}=|d\sin^2\theta+f\cos^2\theta|^2+|(d+f)\sin\theta\cos\theta|^2, \qquad (15)$$

$$I_{E_2}=I_{E_2,\parallel}+I_{E_2,\perp}=|2h\sin\theta\cos\theta|^2+|h|^2. \qquad (16)$$

Base on the above discussion, polarized Raman spectra can be applied to determine the crystal orientation of 2D materials.

Supplementary Note 5. Photoresponsivity (R), detectivity (D_M^* and D_C^*), external quantum efficiency (EQE) and photogain (G) analysis

To quantitatively confirm the device performance, photoresponsivity (R), detectivity (D_M^* and D_C^*), external quantum efficiency (EQE) and photo-gain (G) are the most important figure of merits. Photoresponsivity (R) can be calculated using Supplementary Equation 17 where I_{ph} is the photocurrent, P is the illuminated laser power density and A is the effective device area, A=15 μm^2. The photocurrent is measured under V_{DS} = 1.0 V, V_{GS} = 0 V, and laser power density is 19.1 $mWcm^{-2}$.

$$R=\frac{I_{ph}}{PA}. \quad (17)$$

The detectivity measures the device ability to respond to weak incident signals[5], which can be calculated through Supplementary Equation 18 based on photoresponse and noise current results, here we define the detectivity extracted from this method to be the measured detectivity (D_M^*):

$$D_M^*=\frac{\sqrt{A}}{i_N}R=\frac{\sqrt{A}}{i_{noise}/\sqrt{\Delta f}}R, \quad (18)$$

where A is the effective device area, Δf is the integration time, i_N is noise current density, i_{noise} is the noise current and R is the responsivity. The noise current i_{noise} is related with noise equivalent power (NEP):

$$NEP=\frac{i_{noise}}{R}. \quad (19)$$

The noise spectral density of the device is measured as shown in Supplementary Figure 23a and 23b at room temperature and 77 K, respectively, the noise at 0 V drain bias is lower than that of at 1.0 V drain bias, and the noise is dominated by flicker (*1/f*) noise which is originated from fluctuations of local electronic states. For our photocurrent measurements, the sampling frequency is much higher than 1 Hz, so the noise in our device should be flicker (*1/f*) noise, which is analogous to previous works about the 2D materials-based devices[6]. When extracting the D_M^* value, the drain bias is at 1.0 V, the integration time is Δf=1000Hz, and the corresponding noise current density is 4.55×10^{-11} $A\sqrt{Hz}^{-1}$ and 3.58×10^{-11} $A\sqrt{Hz}^{-1}$ at room temperature and 77 K, respectively.

In the field of 2D materials-based devices, some works have implemented the dark current to calculate the detectivity, in this algorithm, the noise current density is replaced by Supplementary Equation 20:

$$i_N=\sqrt{2qI_{dark}\Delta f}, \quad (20)$$

where q is the electron charge, and I_{dark} is the dark current of the device without laser illumination [5]. Then implement Supplementary Equation 20 into Supplementary Equation 18, the detectivity can be calculated by Supplementary Equation 21, here we refer this detectivity as the calculated detectivity (D_C^*):

$$D_C^*=\sqrt{\frac{A}{2qI_{dark}}}R. \quad (21)$$

Based on equation Supplementary Equation 21, it's noted that Δf is removed, so in most works, Δf can be ignored to extract D_C^* data. To make it more convenient to compare the device performances with previous works, the calculated detectivity (D_C^*) is also summarized in Supplementary Figure 11 for the Te-based device. The dark current is measured for many times before laser illumination, the drain bias is from -1.0 V to 1.0 V, as shown in Supplementary Figure 23c and 23d at room temperature and 77 K, respectively. The dark current can lead to overestimated detectivity comparing with the noise density spectral, as shown in Supplementary Figure 11, the measured detectivity D_M^* is about 1-2 magnitude lower than the calculated detectivity D_C^*. Here the D_M^* value is more accurate to characterize the device performances.

External quantum efficiency (EQE) can be calculated through Supplementary Equation 22, which measures the ratio of photogenerated carriers number over incident photon numbers in one second.

$$EQE=\frac{hc}{q\lambda}R, \quad (22)$$

where R is the photoresponsivity, h is the Plank constant, and λ and c is the wavelength and velocity of incident laser respectively.

Photogain characterizes the ratio between the photo carrier's life time (τ_l) and transit time (τ_T) through the channel, which can be calculated from Supplementary Equation 23 and 24.

$$\tau_{\mathrm{T}}=\frac{L^2}{\mu V_{\mathrm{DS}}}, \quad (23)$$

$$G=\frac{\tau_{\mathrm{l}}}{\tau_{\mathrm{T}}}, \quad (24)$$

where L is the channel length, μ is the mobility and V_{DS} is the drain bias. From transient photoluminescence decay curve in Supplementary Figure 10, the carrier's life time τ_{l}=~900 ns. For our high quality Te, the high mobility and narrow channel length is beneficial for fast transit time $\tau_{\mathrm{T}}=3.838\times10^{-10}$ s, and the long carrier's life time τ_{l} is a result of shallow trap at defects inside the Te crystal due to band tail states. The photogain is at a high level of ~2300, which is not a result of trap-induced photogating as we have discussed in the manuscript.

5.1 Calculation example

Here we have performed the photocurrent measurements under the illumination with the wavelength of 3.0 μm at room temperature for example to calculate the above figure of merits. From Figure 2b, under 1.0 mW laser illumination, the net photocurrent at 1.0 V drain bias is 1.6883 μA. The radius of the laser spot is 1000 μm, and the channel length between electrode 2 and 4 in Figure 1c, is ~6 μm, and the channel width is ~2.5 μm. Then the photoresponsivity is calculated by following:

$$R=\frac{I_{\mathrm{ph}}}{PA}=\frac{1.6883\times10^{-6}\ \mathrm{A}}{1.0\times10^{-3}\ \mathrm{W}\times\frac{6\times2.5}{\pi\times1000^2}}=353.5967\ \mathrm{AW}^{-1}. \quad (25)$$

The noise current density is $4.55\times10^{-11}\ \mathrm{A}\sqrt{\mathrm{Hz}}^{-1}$, so the measured detectivity of 3.0 μm illumination is

$$D_{\mathrm{M}}^{*}=\frac{\sqrt{A}}{i_{\mathrm{N}}}R=\frac{\sqrt{6\times10^{-4}\times2.5\times10^{-4}\ \mathrm{cm}^2}}{4.55\times10^{-11}\ \mathrm{A}\sqrt{\mathrm{Hz}}^{-1}}\times353.5967\ \mathrm{AW}^{-1}$$
$$=3.0098\times10^{9}\ \mathrm{cm\ W^{-1}Hz^{1/2}}. \quad (26)$$

The dark current is $I_{\mathrm{dark}}=1.066\ \mu\mathrm{A}$, then the calculated detectivity of 3.0 μm illumination is

$$D_{\mathrm{C}}^{*}=\sqrt{\frac{A}{2qI_{\mathrm{dark}}}}R=\sqrt{\frac{6\times10^{-4}\times2.5\times10^{-4}\ \mathrm{cm}^2}{2\times1.6\times10^{-19}\ \mathrm{C}\times1.066\times10^{-6}\mathrm{A}}}\times353.596\ \mathrm{AW}^{-1}$$
$$=2.3448\times10^{11}\ \mathrm{cm\ W^{-1}Hz^{1/2}}. \quad (27)$$

The *EQE* of 3.0 μm illumination is

$$EQE=\frac{hc}{q\lambda}R=\frac{6.626\times10^{-34}\ \mathrm{Js}\times3.0\times10^{8}\ \mathrm{ms}^{-1}}{1.6\times10^{-19}\ \mathrm{C}\times3\times10^{-6}\ \mathrm{m}}\times353.5967\ \mathrm{AW}^{-1}=146.4332 \quad (28)$$

Supplementary Note 6. Scattering theory

6.1 Scattering parameters related with polarization

Analogous to scattering conditions such as foggy and cloudy weather, colloid and emulsion media, when the light passes through the scattering media in our experiments, it will be scattered or absorbed. The angle between incident wave vector $\mathbf{k_i}$ and scatter wave vector $\mathbf{k_s}$ is θ_s (Supplementary Figure 24). Since the scattered light is highly polarized, here we focus on several parameters for the scattered light.

1) Scattering amplitude

$$A_s(\theta_s)=A_{s,\perp}(\theta_s)+A_{s,\parallel}(\theta_s). \quad (29)$$

$A_{s,\perp}(\theta_s)$ is the amplitude perpendicular to the scatter plane, and $A_{s,\parallel}(\theta_s)$ is the amplitude parallel to the scatter plane. Based on Supplementary Equation 29, the intensity of scattered light is angle-dependent, as defined by Supplementary Equation 30:

$$I_s(\theta_s)=I_{s,\perp}(\theta_s)+I_{s,\parallel}(\theta_s)=|A_{s,\perp}(\theta_s)|^2+|A_{s,\parallel}(\theta_s)|^2. \quad (30)$$

2) Polarization degree of scattered light

$$P_s=\frac{I_{s,\perp}(\theta_s)-I_{s,\parallel}(\theta_s)}{I_{s,\perp}(\theta_s)+I_{s,\parallel}(\theta_s)}. \quad (31)$$

3) Scattering cross sections

The scattering cross section is used to describe the probability of light being scattered by macroscopic or microscopic particles in the scattering media, which is related with the incident wavelength, shape and size of the particles, and this parameter also shows strong angle-dependent. The scattering cross section can be calculated from scattered light intensity:

$$\sigma_{sc}=\frac{1}{k^2}\int I_s(\theta_s)\sin\theta d\theta. \quad (32)$$

6.2 Rayleigh scattering

When the scattering particle size is smaller than the incident wavelength, Rayleigh scattering is dominant. The scattering media can be treated as a porous material, where the solid part and air exhibit strong contrast in refractive index. After passing through the scattering media, the transport direction of light is changed, here

we neglect the influence of coherence as the scattering position is randomly distributed. Then the resulting scattering intensity can be calculated by the sum of the squares of amplitudes, which is also angle-dependent, as shown in Supplementary Equation 33:

$$I_{\mathrm{RL}}=I_0\frac{1+\cos^2\theta}{2R^2}\left(\frac{2\pi}{\lambda}\right)^4\left(\frac{n^2-1}{n^2+2}\right)^2\left(\frac{d}{2}\right)^6, \qquad (33)$$

where I_0 is the intensity of unpolarized incident light, λ is the wavelength of incident light, n is the refractive index of the scattering particle, R is the distance from incident source to the scattering particle, d is the diameter of the scattering particle.

6.3 Mie scattering

When the scattering particle size is similar to the incident wavelength, the scattering behavior is explained by Mie scattering. The scattered light intensity can be calculated by Supplementary Equation 34:

$$I_{\mathrm{M}}=I_0\frac{\lambda^2}{2\pi^2d^2}[I_{\mathrm{M},\perp}(\theta)+I_{\mathrm{M},\parallel}(\theta)]. \qquad (34)$$

Then the polarization degree of Mie scattering can be acquired directly:

$$P_{\mathrm{M}}=\frac{I_{\mathrm{M},\perp}(\theta)-I_{\mathrm{M},\parallel}(\theta)}{I_{\mathrm{M},\perp}(\theta)+I_{\mathrm{M},\parallel}(\theta)}. \qquad (35)$$

Supplementary Note 7. Fundamentals of polarimeters

The most important part for polarization imaging device is the polarimeters. For each pixel, the polarization signal is detected through a polarization filter and then recorded as variation in intensity [7-9]. Generally, the polarization state of the incident signal is quantified by a Stocks vector, and each element in the Stocks vector is related with intensity at particular directions [7-9].

$$\vec{S}(x,y)=\begin{bmatrix}S_0(x,y)\\S_1(x,y)\\S_2(x,y)\\S_3(x,y)\end{bmatrix}=\begin{bmatrix}I_{0^\circ}(x,y)+I_{90^\circ}(x,y)\\I_{0^\circ}(x,y)-I_{90^\circ}(x,y)\\I_{45^\circ}(x,y)-I_{135^\circ}(x,y)\\I_{\mathrm{R}}(x,y)-I_{\mathrm{L}}(x,y)\end{bmatrix}, \tag{36}$$

where $I_{0^\circ}(x,y)$, $I_{90^\circ}(x,y)$, $I_{45^\circ}(x,y)$, $I_{135^\circ}(x,y)$ refers to the linear polarized intensity along 0°, 90°, 45° and 135° direction, $I_{\mathrm{R}}(x,y)$ and $I_{\mathrm{L}}(x,y)$ refers to the right circular and left circular polarized intensity. So, S_1 and S_2 characterize the affinity of linear polarization, and S_3 characterizes the affinity of circular polarization.

The polarization figure of merits can be extracted from the Stocks vector. The angle of linear polarization is defined by Supplementary Equation 37.

$$\theta_{\mathrm{linear}}=\frac{1}{2}\arctan\left(\frac{S_2}{S_1}\right). \tag{37}$$

The total degree of polarization is defined by Supplementary Equation 38.

$$TDoP=\frac{\sqrt{S_1^2+S_2^2+S_3^2}}{S_0}. \tag{38}$$

The degree of linear polarization is defined by Supplementary Equation 39.

$$DoLP=\frac{\sqrt{S_1^2+S_2^2}}{S_0}. \tag{39}$$

The degree of circular polarization is defined by Supplementary Equation 40.

$$DoCP=\frac{S_3}{S_0}. \tag{40}$$

Here we focus on the linear polarization imaging applications, so the imaging ability is measured by calculating *DoLP* for each incident wavelength. Our imaging mechanism is based on the division-of-focal-plane polarimeter (*DoFP*) structure, the schematic for this structure is shown in Supplementary Figure 22. Under strong scattering environments, the incident signal is high polarized along one direction, and

the intensity is weak along the perpendicular direction. In our experiments, the polarization along 0°, 90°, 45° and 135° directions are measured by rotating a half wave plate, the intensity for each pixel is detected by changing the location of our tellurium device through *x*-axis and *y*-axis step motor. So, we have acquired four imaging results along the selected direction, and then we have calculated the *DoLP* based on above equations. Comparing with original imaging results, *DoLP* imaging results can provide us with more clear imaging quality. The imaging contrast is stronger when *DoLP* is larger, which is related with the absorption anisotropic ratio of the sensing material, so *DoLP* is wavelength sensitive (Figure 4d-4e). For materials without polarization sensitivity, *DoLP* is almost zero and no clear polarization imaging can be realized (Figure 4c).

Supplementary Note 8. Low temperature detection characters in the visible range

Low temperature characters (77 K) are also measured, from 0.52 μm to 3.0 μm. Under 77 K temperature, the dark current can be much lower comparing with that of at room temperature (Supplementary Figure 23d), which is beneficial to realize higher detectivity. In addition, the net photocurrent is defined by Supplementary Equation 41:

$$I_{\mathrm{ph\ net}} = I_{\mathrm{ph}} - I_{\mathrm{dark}}, \qquad (41)$$

which is also increased, comparing with the room temperature case. As a result, the photoresponsivity, detectivity and photogain are much higher at low temperature.

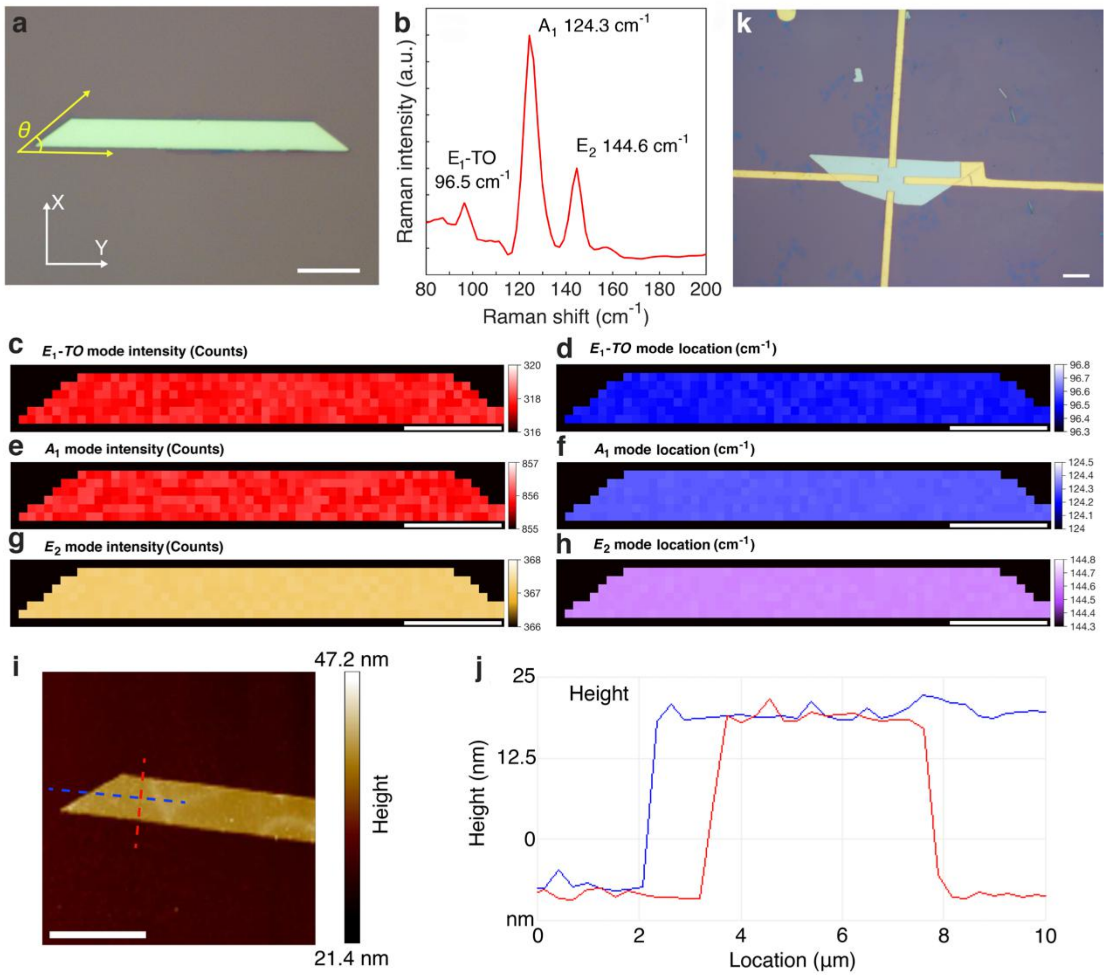

Supplementary Figure 1. Crystal quality characterization. (a) The optical image of tellurium nanoflake. The y axis is along the longer side of the flake, and x axis is perpendicular to the longer side of the flake. θ is the angle between longer side of the crystal and incident light polarization direction for Raman simulation. Scale bar, 10 μm. (b) Raman line shape of Te. E_1-TO, A_1 and E_2 mode are located at 96.5 cm^{-1}, 124.3 cm^{-1}, and 144.6 cm^{-1}, respectively. The FWHM for A_1 peak is 7.6 cm^{-1}, which indicates the high crystal quality of our sample. (c)-(h) Raman mapping results. (c) E_1-TO mode intensity, (d) E_1-TO mode peak location, (e) A_1 mode intensity, (f) A_1 mode location, (g) E_2 mode intensity, (h) E_2 mode peak location. Scale bars, 10 μm. (i)-(j) AFM results of synthesized tellurium nanoflake. (i) The AFM image of the tellurium crystal, scale bar, 10 μm, (j) shows the height distribution of tellurium along the red and blue dashed line in (i), respectively. The thickness of the Te crystal is ~27.5 nm. (k) Optical images of the Te FET device for anisotropic electrical performance measurements. The channel width is ~10 μm for both directions. Scale

bar, 10 μm.

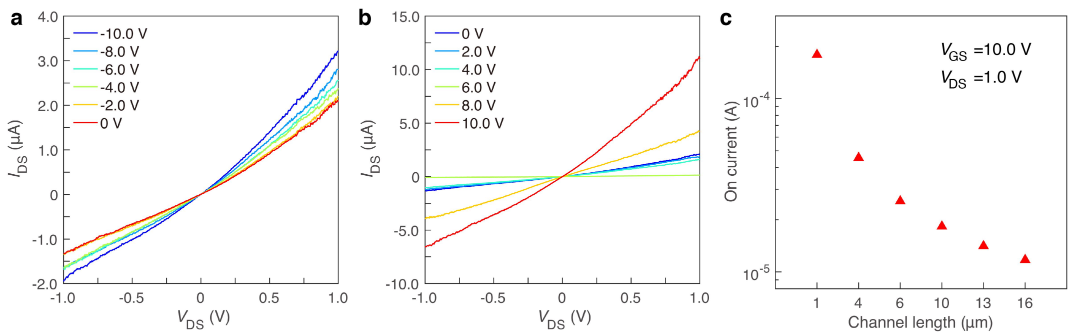

Supplementary Figure 2. Electronic performances under different gate bias. (a)-(b) Drain current under different gate bias from -10.0 V to 10.0 V, for the 16 μm channel length. (c) Maximum on current at 10.0 V gate bias for different channel length, the drain bias is 1.0 V.

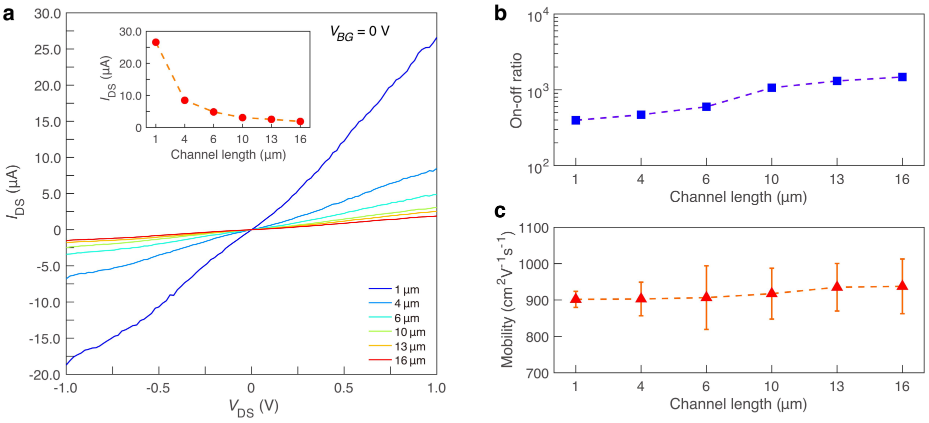

Supplementary Figure 3. Electronic characters for different channel length. (a) Drain current at different channel length, the gate bias is fixed at 0 V, the inset shows the drain current under 1.0 V drain bias, the drain current is increasing with narrower channel length. (b) The relationship between on-off ratio and channel length, calculated from curves in Figure 1d, the on-off ratio is slightly decreasing with narrower channel length. (c) Field effect carrier mobility for different channel length, calculated from curves in Figure 1d, which changes slightly. The error bar is calculated based on transfer curves in Figure 1d.

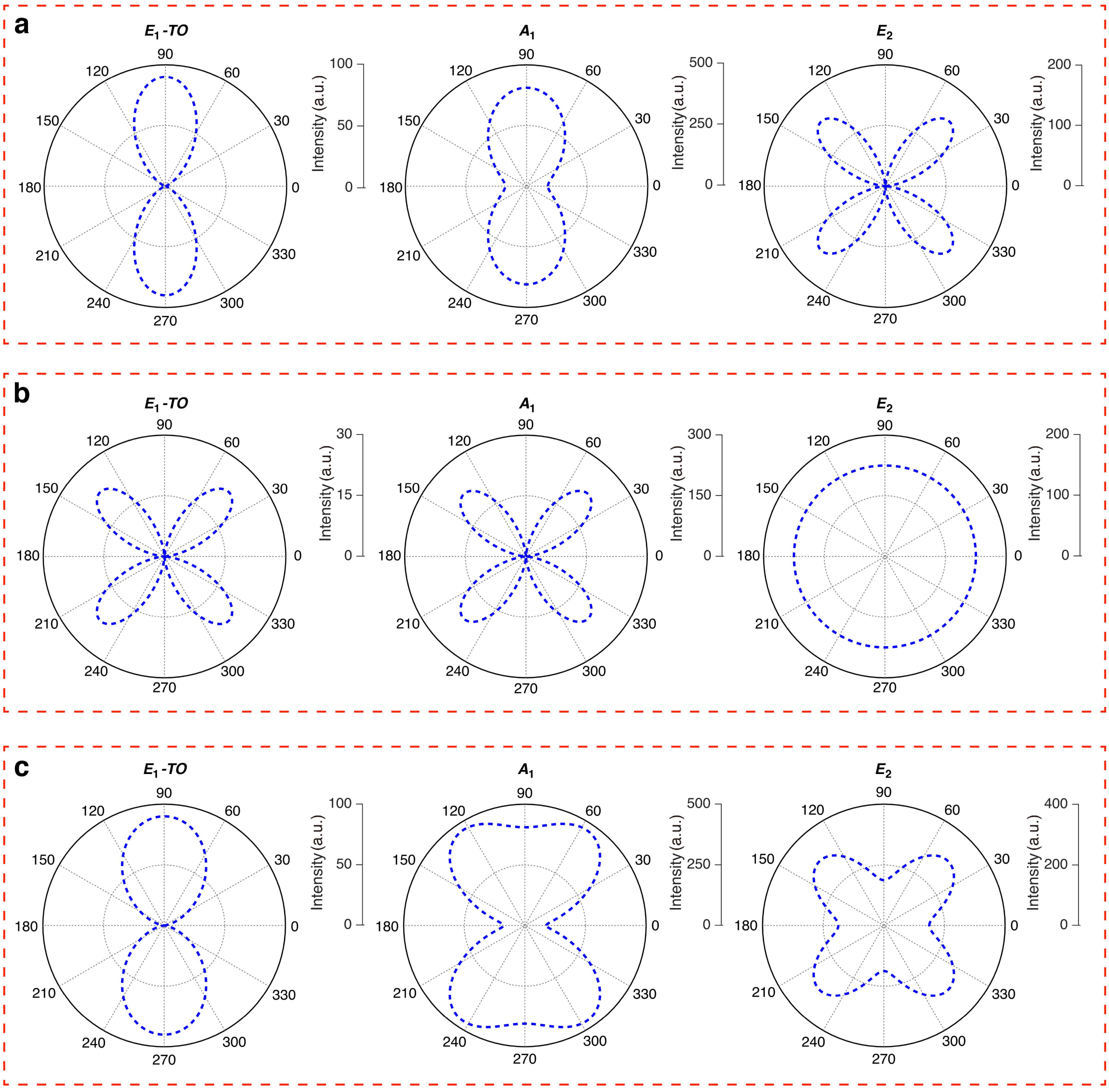

Supplementary Figure 4. Raman intensity calculation results. (a) The scattering polarization direction is parallel to the incident polarization direction. (b) The scattering polarization direction is perpendicular to the incident polarization direction. (c) For the condition where the scattering light is unpolarized, we consider two directions where the scattering direction is parallel and perpendicular to the incident polarization direction.

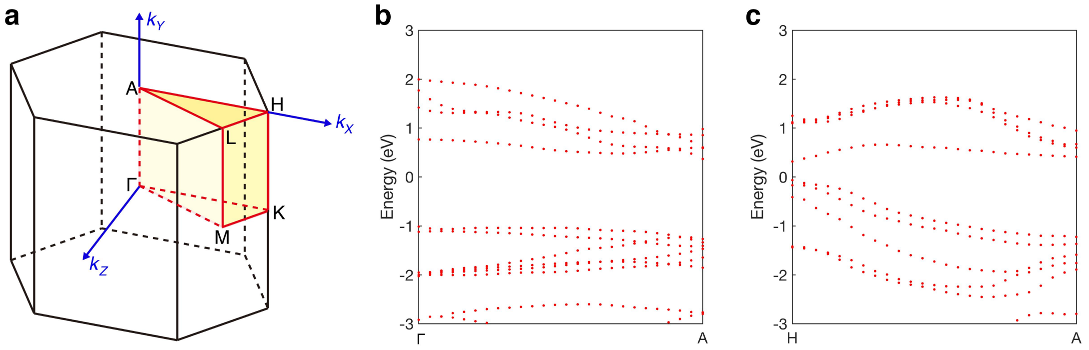

Supplementary Figure 5. Band structure of tellurium by first principle calculations. (a) Brillouin zone of bulk tellurium. (b) and (c) are band structure along *Γ*-A and H-A paths, which are corresponding to y and x direction respectively. The difference in band structure is the origin of anisotropic characters.

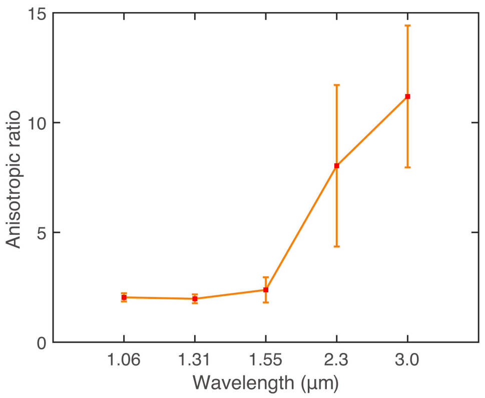

Supplementary Figure 6. Anisotropic ratio for absorption spectra at several selected wavelengths. The anisotropic ratio is enhanced to be more than 8 from 2.3 μm to 3.0 μm, which is beneficial for polarized photodetection applications. The error bar is calculated based on absorption measurements.

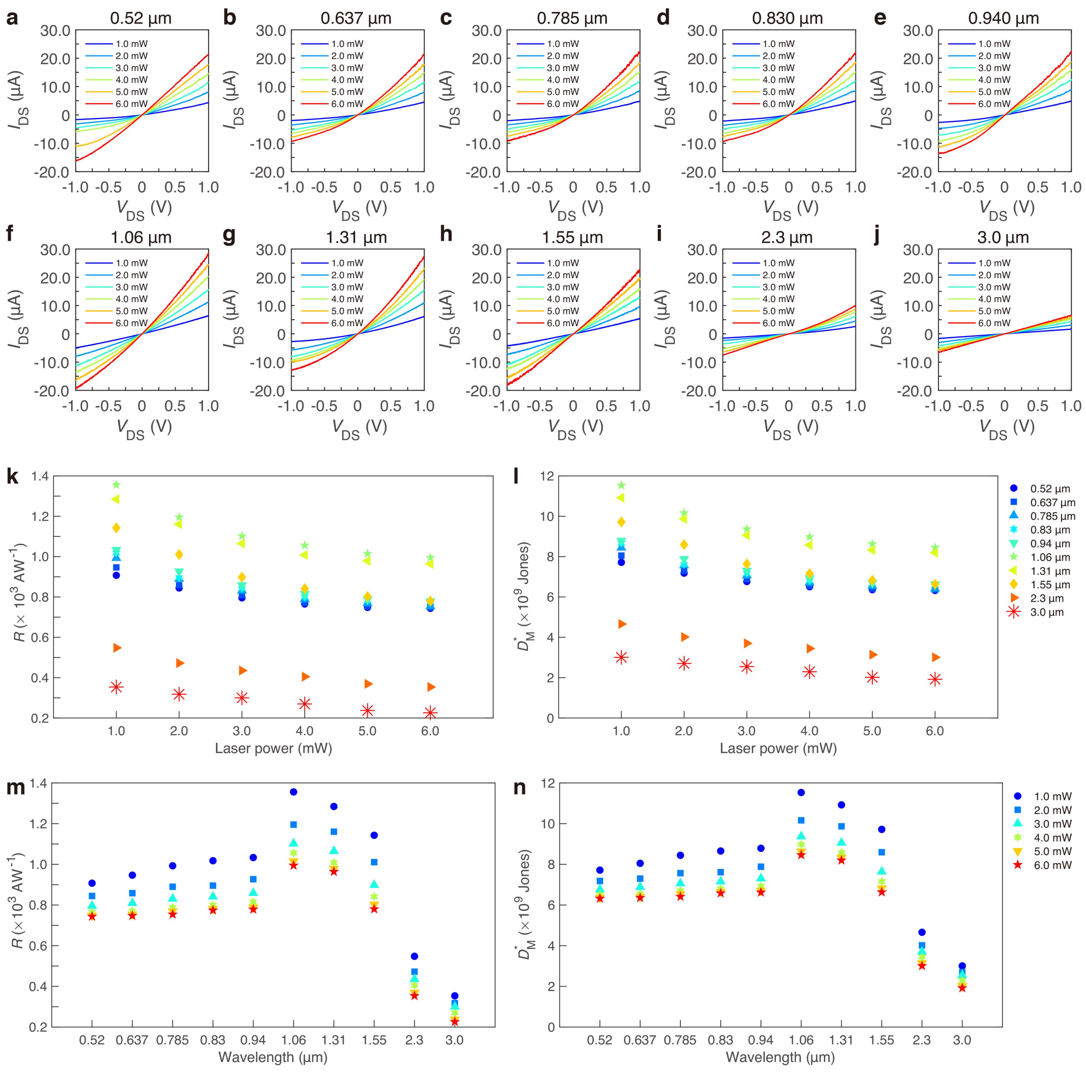

Supplementary Figure 7. Unpolarized broadband optoelectronic responses for tellurium nanoflake device at room temperature (300 K). (a)-(j) Photocurrent for 0.52 μm, 0.637 μm, 0.785 μm, 0.83 μm, 0.94 μm, 0.106 μm, 0.131 μm, 0.155 μm, 2.3 μm, and 3.0 μm illumination, respectively, as a function of laser power and drain bias, the gate bias is 0 V. (k)-(l) R and D_M^* as a function of laser power for different wavelengths, respectively, the drain bias is fixed at 1.0 V, the gate bias is 0 V. (m)-(n) R and D_M^* as function of wavelength under different illumination laser powers at room temperature.

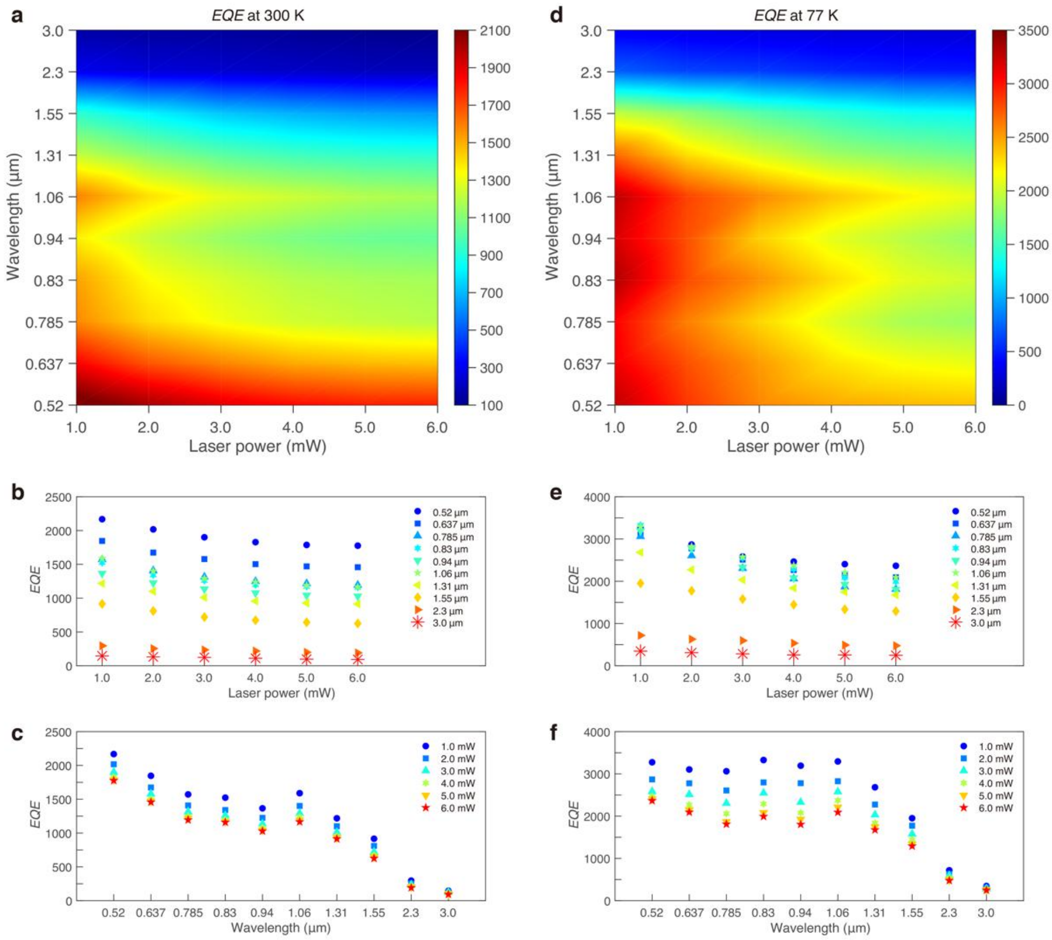

Supplementary Figure 8. (a)-(c) *EQE* results at room temperature. (a) Pseudo-color mapping image of *EQE*. (b) *EQE* as a function of illumination laser power for different wavelengths at room temperature. (c) *EQE* as a function of wavelength under different laser powers at room temperature. (d)-(f) *EQE* results at low temperature. (d) Pseudo-color mapping image of *EQE*. (e) *EQE* as a function of illumination laser power for different wavelengths at 77 K temperature. (f) *EQE* as a function of wavelength under different laser powers at 77 K temperature. The performance under 77 K is higher.

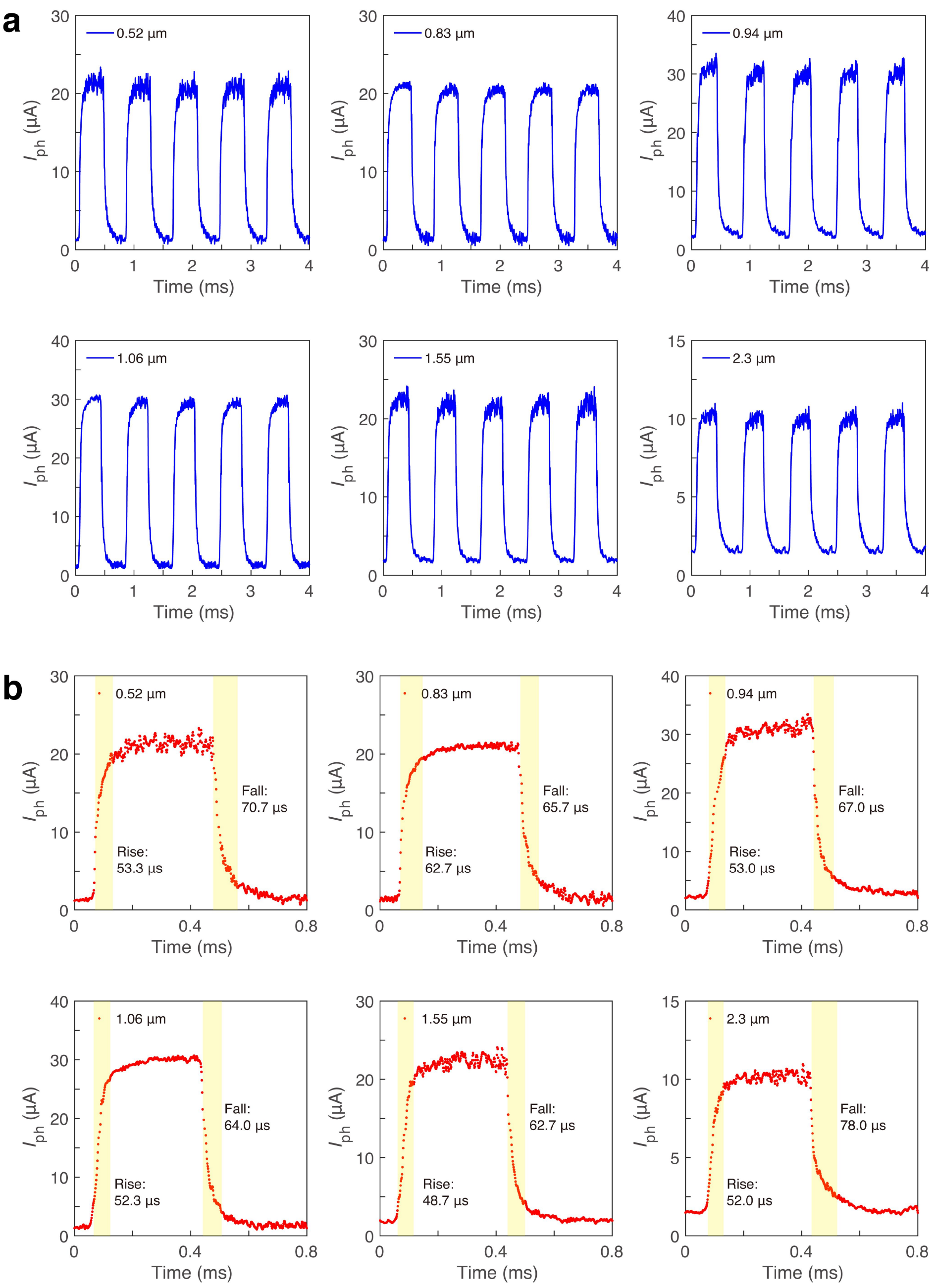

Supplementary Figure 9. Response time under different illumination wavelengths at room temperature. The drain bias is fixed at 1.0 V and the laser power is fixed at 6.0 mW. (a) Photocurrent responses in five cycles. (b) Photocurrent rise time and fall time calculated from curves in (a).

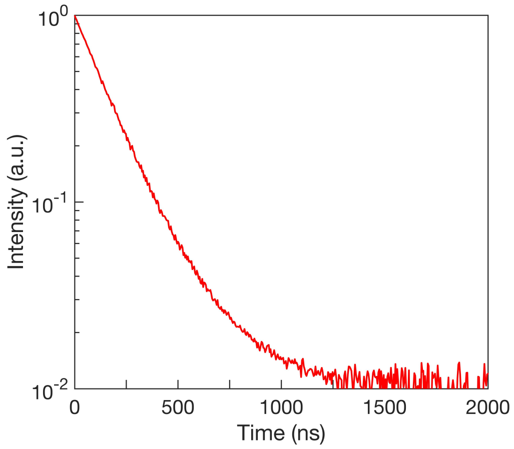

Supplementary Figure 10. Transient photoluminescence decay curves of Te. The carrier lifetime is ~900 ns. Based on the carrier lifetime and transit time, the photogain can be calculated to be ~2300.

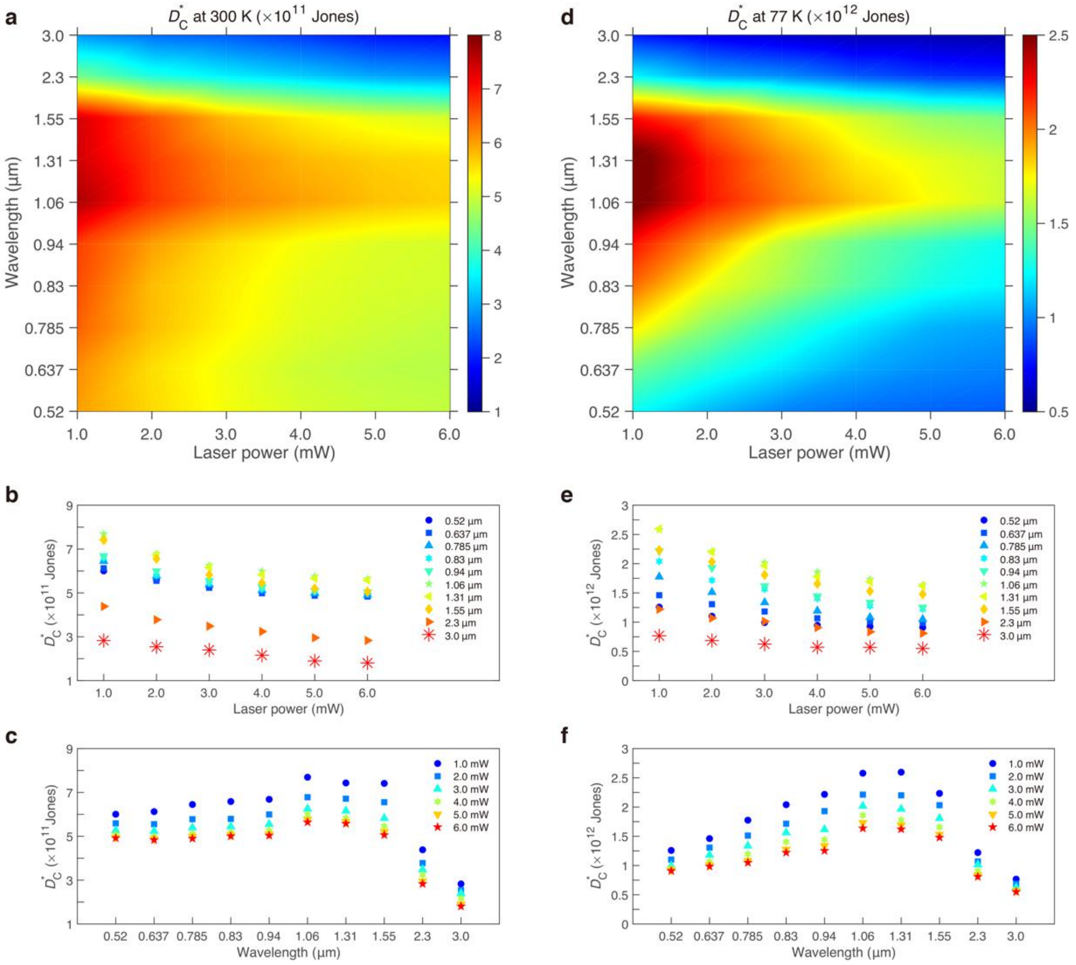

Supplementary Figure 11. The calculated detectivity (D_C^*) results. (a)-(c) D_C^* at room temperature. (a) Pseudo-color mapping results of D_C^*. (b) D_C^* as a function of laser power for different wavelengths at room temperature. (c) D_C^* as a function of wavelength under different laser powers at room temperature. (d)-(f) D_C^* at 77 K temperature. (a) Pseudo-color mapping results of D_C^*. (b) D_C^* as a function of laser power for different wavelengths at 77 K temperature. (c) D_C^* as a function of wavelength under different laser powers at 77 K temperature. D_C^* is about 1-2 magnitude higher than D_M^* due to the lower dark current than the actual noise current.

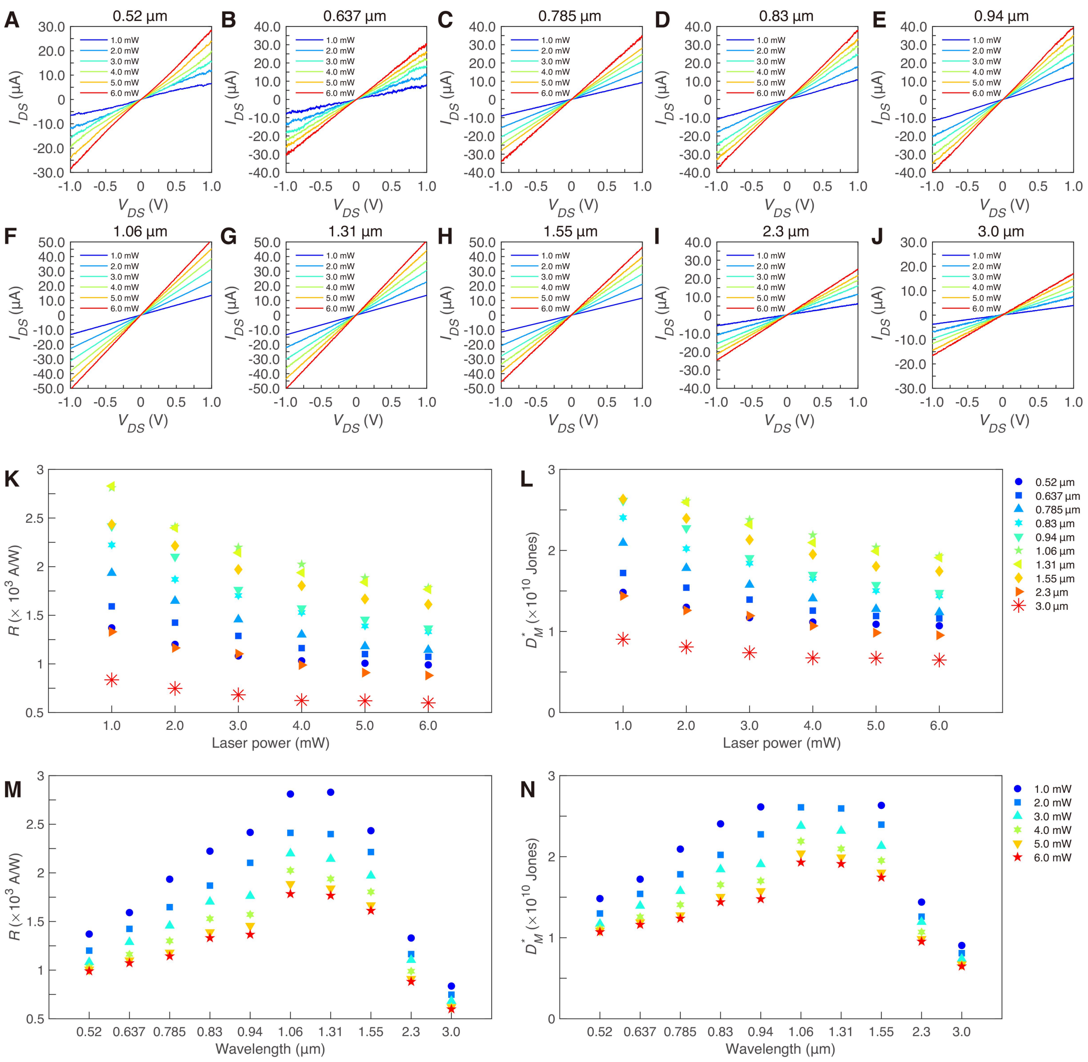

Supplementary Figure 12. Unpolarized broadband optoelectronic responses for tellurium nanoflake device at 77 K. (a)-(j) Photocurrent under illumination of 0.52 μm, 0.637 μm, 0.785 μm, 0.83 μm, 0.94 μm, 0.106 μm, 0.131 μm, 0.155 μm, 2.3 μm, and 3.0 μm, respectively. The gate bias is fixed at 0 V. (k)-(l) R and D_M^* as a function of laser power for different wavelengths, respectively, the drain bias is fixed at 1.0 V, the gate bias is 0 V. (m)-(n) R and D_M^* as function of wavelength under different illumination laser powers at 77 K temperature.

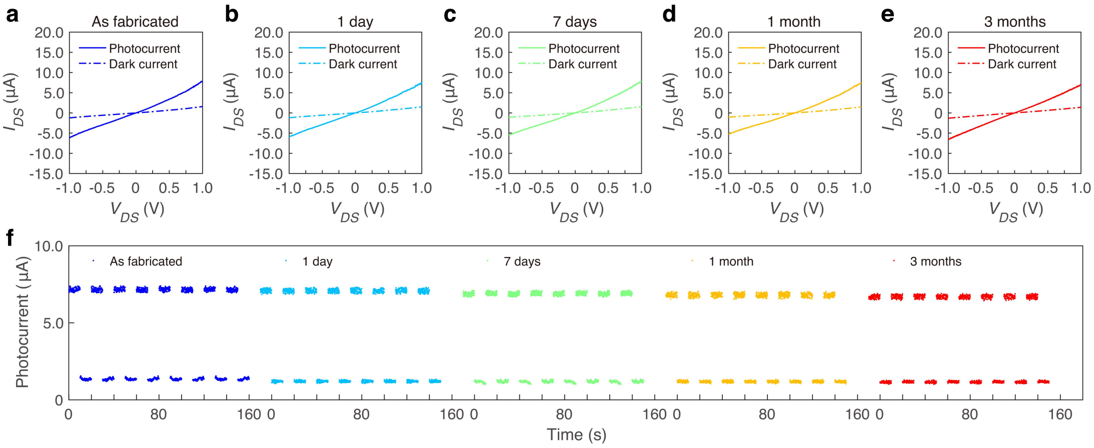

Supplementary Figure 13. Stability of the tellurium device at room temperature. To verify the stability of our device, the selected incident wavelength is 3.0 μm, the laser power is 6.0 mW, and the drain bias is from -1.0 V to 1.0 V. All the measurements are performed at ambient conditions, and the device is also preserved at ambient conditions. (a)-(e) Drain current results for the tellurium device, measured at 5 different time, as fabricated, 1 day later, 7 days later, 1 month later and 3 months later, respectively. The dashed line is the dark current without laser illumination, and the solid line is the photocurrent under 3.0 μm illumination. The device performance is not degraded after 3 months. (f) Photocurrent as a function of time, the drain bias is fixed at 1.0 V, the illumination period is 40 s with duty ratio of 0.5, the high performance of tellurium device is verified, and the stability of tellurium is much better than commonly used anisotropic 2D materials such as black phosphorus and black phosphorus-arsenic.

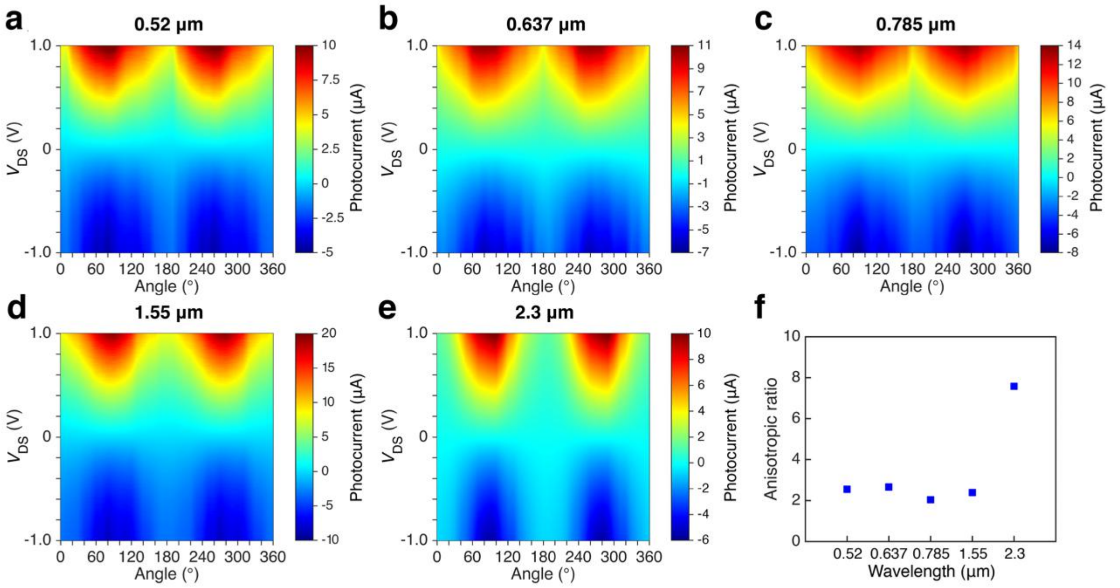

Supplementary Figure 14. Linear polarized photocurrent under various wavelengths. The drain bias is from -1.0 V to 1.0 V, the gate bias is 0, and the incident power is 6.0 mW. (a)-(e) are photocurrent for 0.52 μm, 0.637 μm, 0.785 μm, 1.55 μm and 2.3 μm, respectively. The incident light is linear polarized, and the polarization direction is fixed, it's initially parallel to the *y* axis of the Te device. By rotating the device in a step of 10˚, linear polarized photocurrent curves are acquired. (f) The linear polarized photocurrent anisotropic ratio for the measured five wavelengths, which is defined as the photocurrent along 90˚ over the photocurrent along 0˚. Highest anisotropic ratio of 7.58 is realized under 2.3 μm illumination, which is very high in the field of 2D materials.

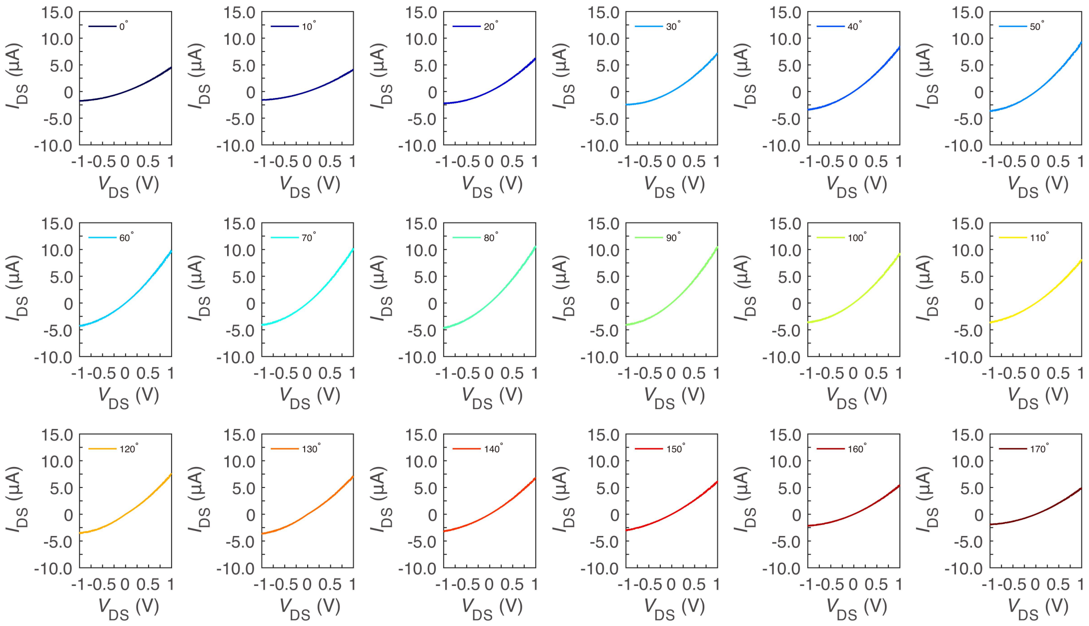

Supplementary Figure 15. Linear polarized photocurrent under 0.52 μm illumination. The incident power is 6.0 mW. The incident light is linear polarized, and the polarization direction is fixed, it's initially parallel to the y axis of the Te device. By rotating the device in a step of 10°, linear polarized photocurrent curves are acquired.

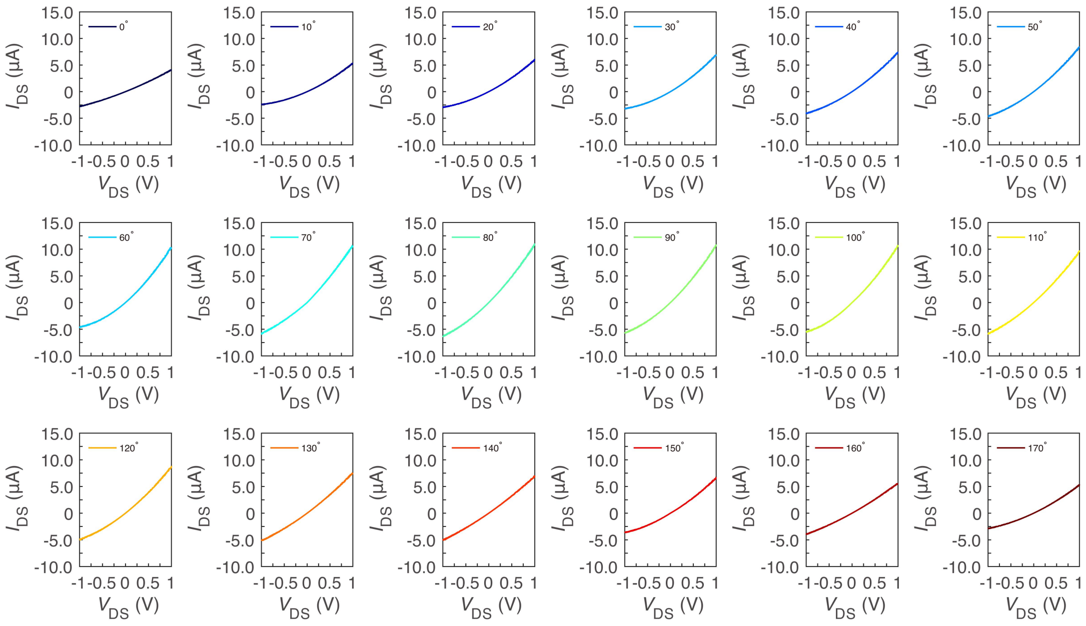

Supplementary Figure 16. Linear polarized photocurrent under 0.637 μm illumination. The incident power is 6.0 mW. The incident light is linear polarized, and the polarization direction is fixed, it's initially parallel to the y axis of the Te device. By rotating the device in a step of 10°, linear polarized photocurrent curves are acquired.

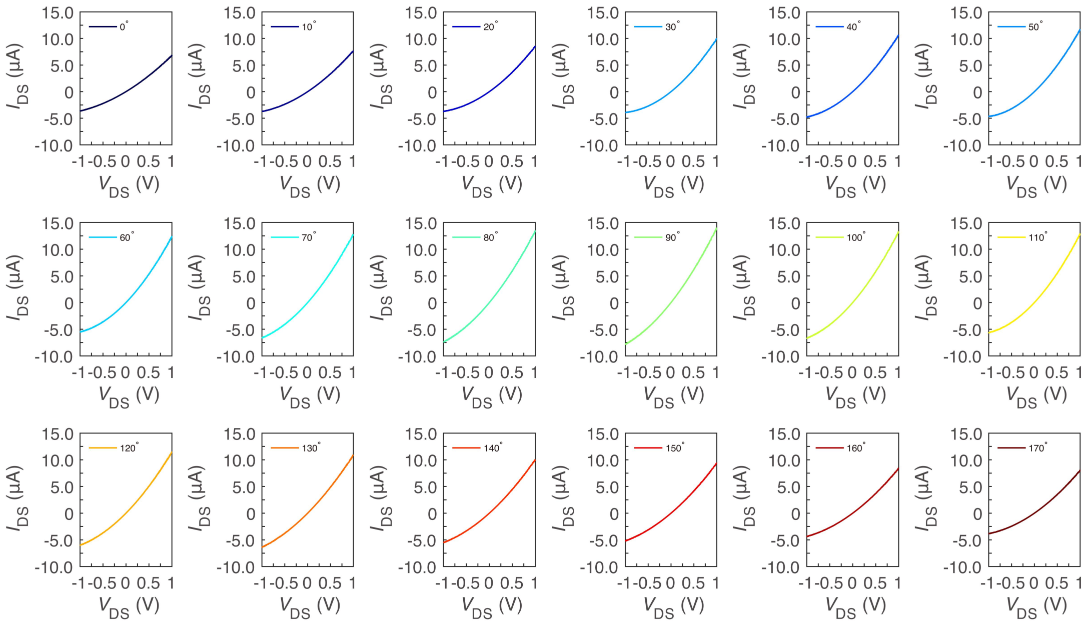

Supplementary Figure 17. Linear polarized photocurrent under 0.785 μm illumination. The incident power is 6.0 mW. The incident light is linear polarized, and the polarization direction is fixed, it's initially parallel to the *y* axis of the Te device. By rotating the device in a step of 10°, linear polarized photocurrent curves are acquired.

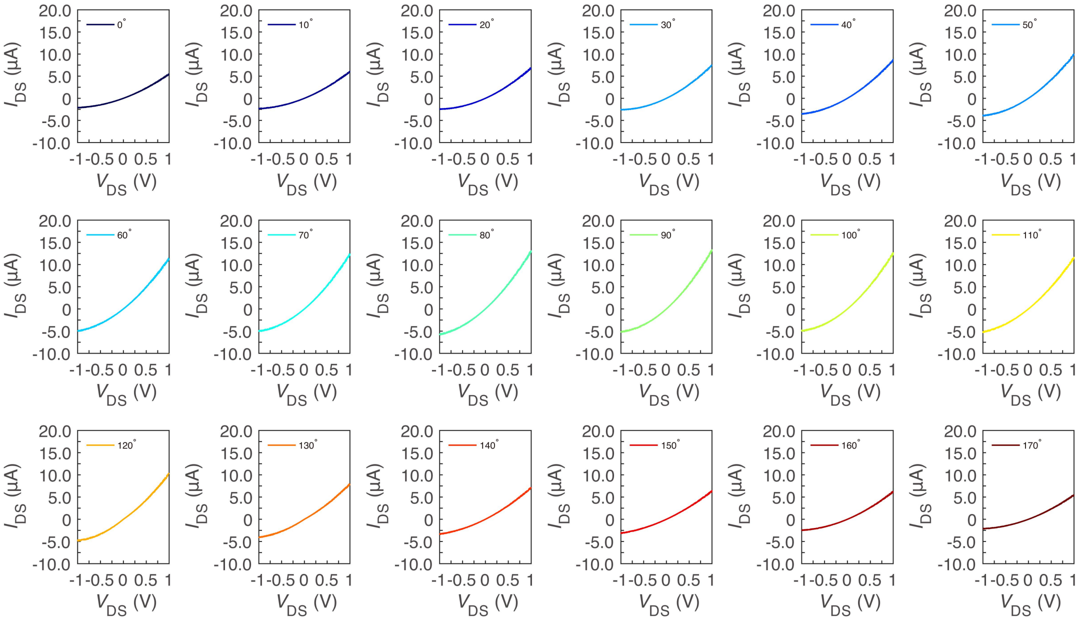

Supplementary Figure 18. Linear polarized photocurrent under 1.55 μm illumination. The incident power is 6.0 mW. The incident light is linear polarized, and the polarization direction is fixed, it's initially parallel to the *y* axis of the Te device. By rotating the device in a step of 10°, linear polarized photocurrent curves are acquired.

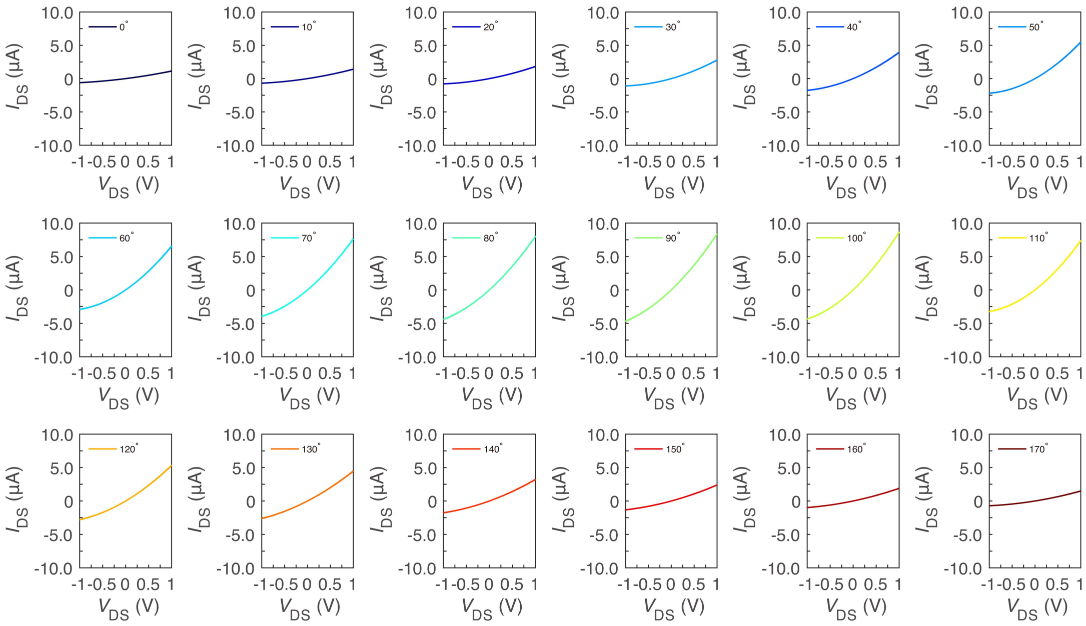

Supplementary Figure 19. Linear polarized photocurrent under 2.3 μm illumination. The incident power is 6.0 mW. The incident light is linear polarized, and the polarization direction is fixed, it's initially parallel to the *y* axis of the Te device. By rotating the device in a step of 10°, linear polarized photocurrent curves are acquired.

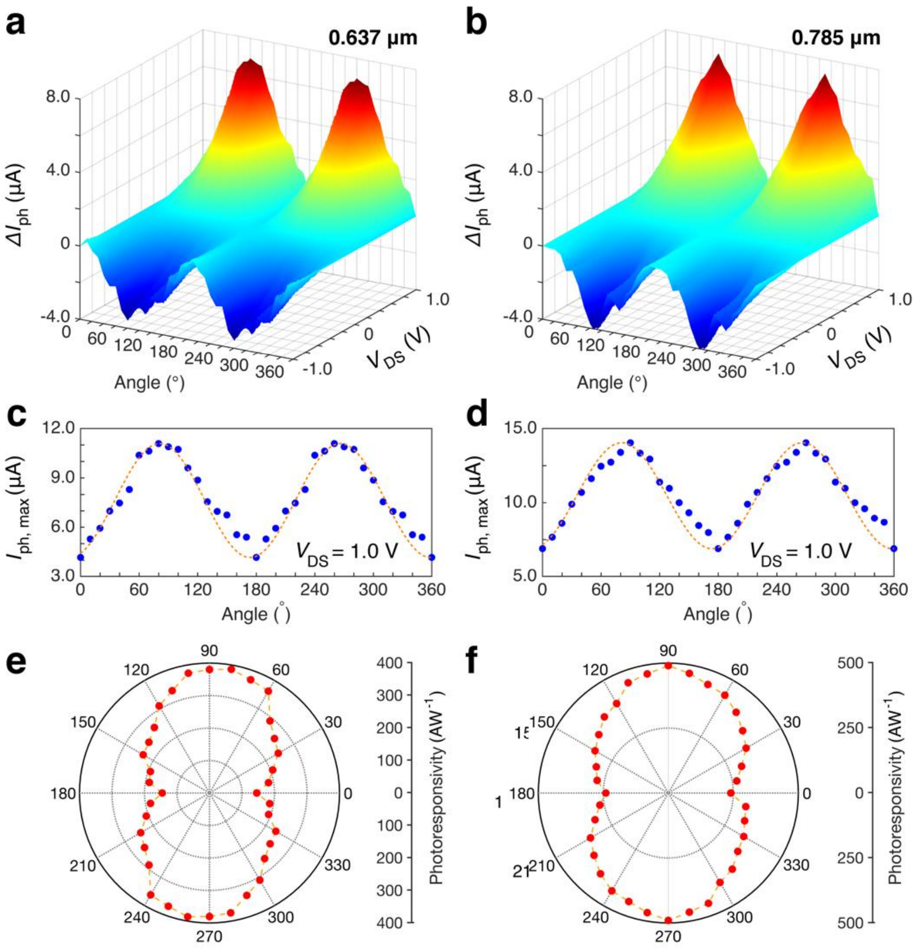

Supplementary Figure 20. Polarized optoelectronic responses for tellurium nanoflake devices. (a)-(b) Net polarized photocurrent ΔI_p for 0.637 μm and 0.785 μm illumination at room temperature, under incident power of 6.0 mW, respectively. (c)-(d) The polarized photocurrent under 1.0 V drain bias for 0.637 μm and 0.785 μm illumination, respectively. The dots are experimental data, and the dashed lines are fitted curves based on sine curve. (e)-(f) The polar diagram of polarized photoresponsivity under 1.0 V drain bias for 0.637 μm and 0.785 μm illumination, respectively, and the anisotropic ratio are 2.66, 2.04 for the two wavelengths.

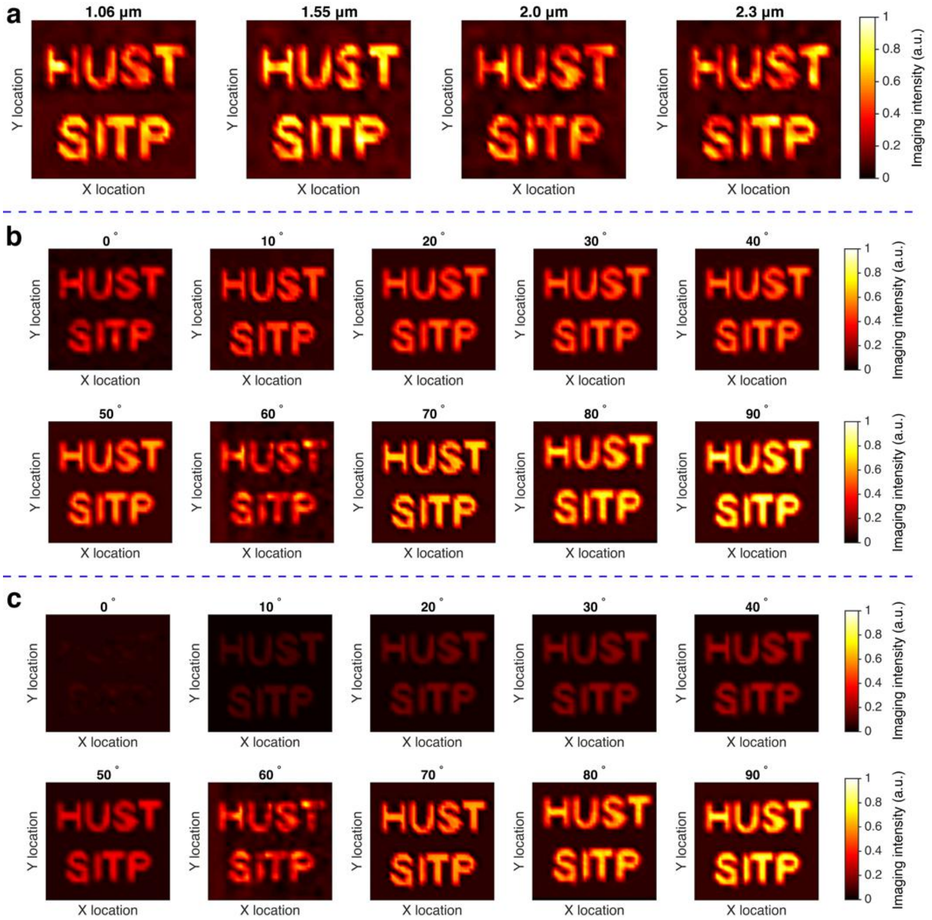

Supplementary Figure 21. Photoimaging experiments. (a) Unpolarized photoimaging experimental results for 1.06 μm, 1.55 μm, 2.0 μm and 2.3 μm, respectively. (b)-(c) Polarized photoimaging under linear polarized illumination of 1.55 μm and 2.3 μm. The linear polarized illumination is acquired through a quarter-wave plate, for our measurements, the illumination polarization direction is fixed to be along the longer side of Te initially (y axis, defined as 0˚), in other words, the quarter-wave plate is never rotated throughout the experiments. By just rotating the device with a step of 10˚, polarized imaging is realized for our Te device, with satisfying high otherness under different orientations, which is related with absorption anisotropy of Te. For 1.55 μm illumination, the detected photocurrent anisotropic ratio is ~2, for 2.3 μm illumination, the detected photocurrent anisotropic ratio is much higher of ~8. The imaging intensity signals are normalized.

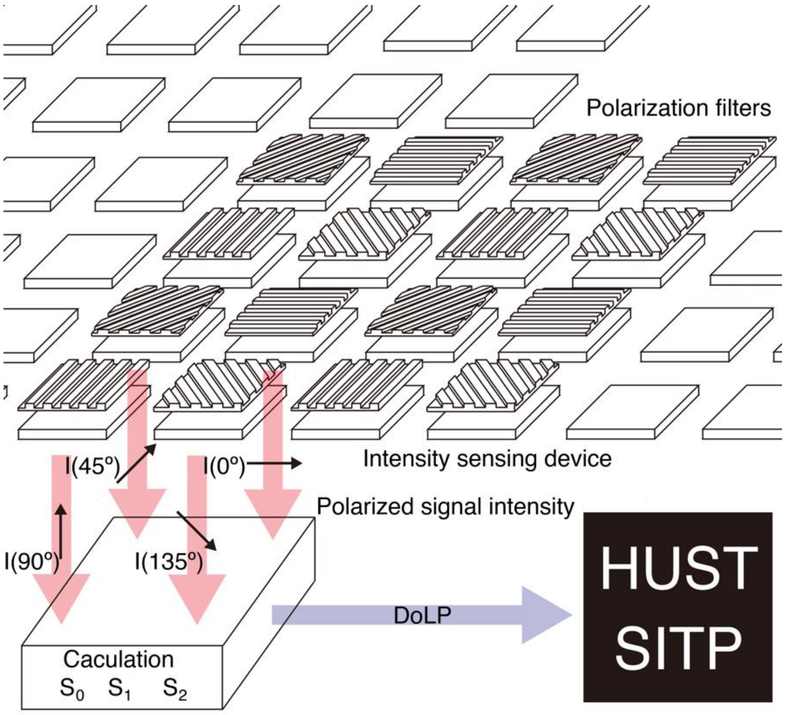

Supplementary Figure 22. Schematic of the division-of-focal-plane polarimeter (DoFP) structure.

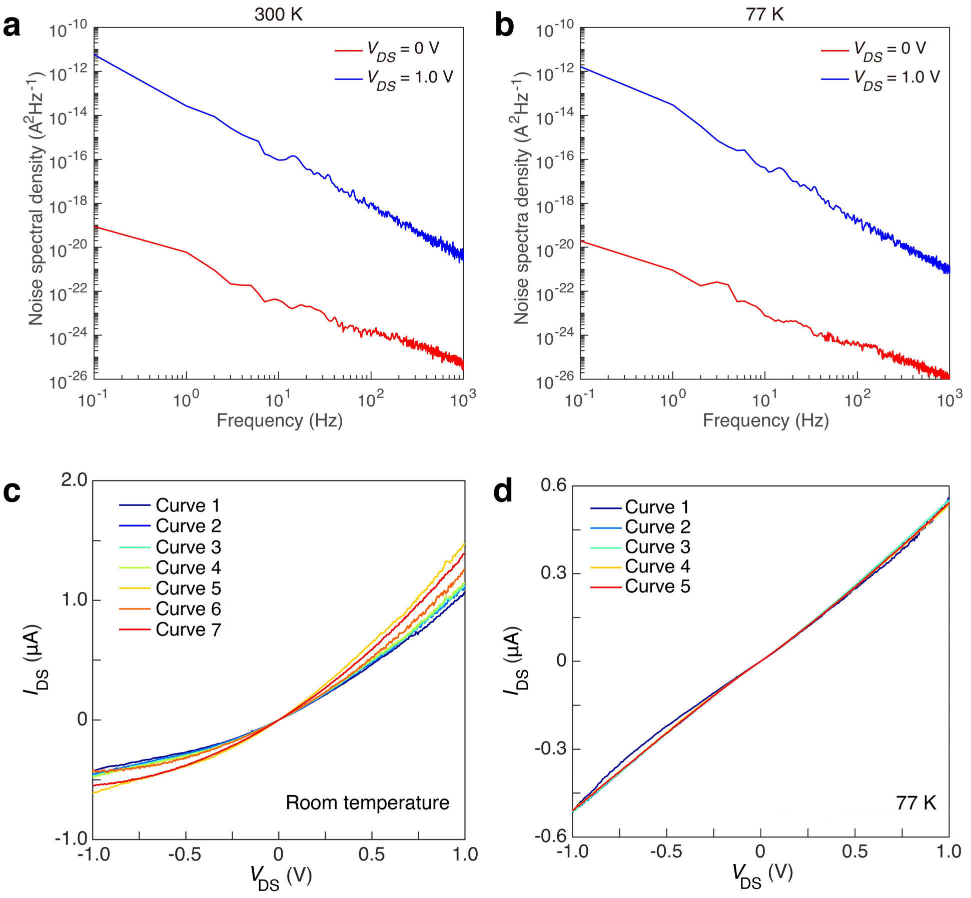

Supplementary Figure 23. (a)-(b) Noise spectral density of the tellurium device at room temperature and 77 K, respectively. The drain bias is fixed at 0 V (red curve) and 1.0 V (blue curve), and the gate bias is 0 V. The noise is dominated by *1/f* noise. (c)-(d) Dark current under drain bias from -1.0 V to 1.0 V at room temperature and 77 K, respectively, the gate bias is 0 V. It's also proved that the dark current is lower than the noise current, which leads to the overestimated calculated detectivity (D_C^*) comparing with the measured detectivity (D_M^*).

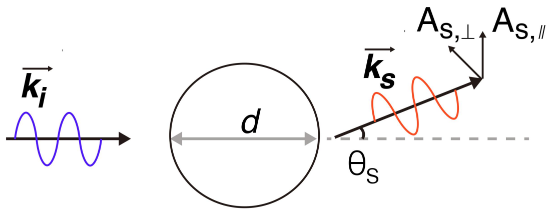

Supplementary Figure 24. Scattering through particles. $\mathbf{k_1}$ is the incident wave vector, $\mathbf{k_2}$ is the scatter wave vector, the scattering angle θ_s is the angle between incident wave vector $\mathbf{k_1}$ and scatter wave vector $\mathbf{k_2}$, and d is the diameter of particles, A denotes absorption. The scattering is highly angle-dependent in theory.

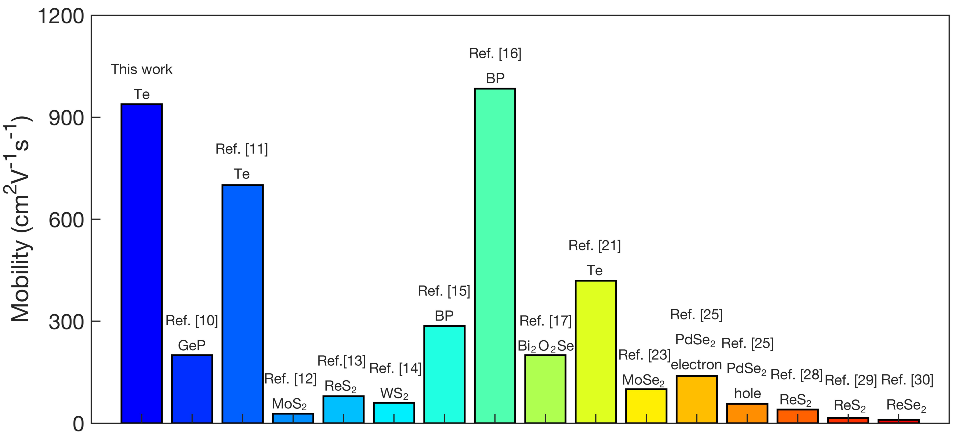

Supplementary Figure 25. Field effect mobility comparison for various 2D materials.

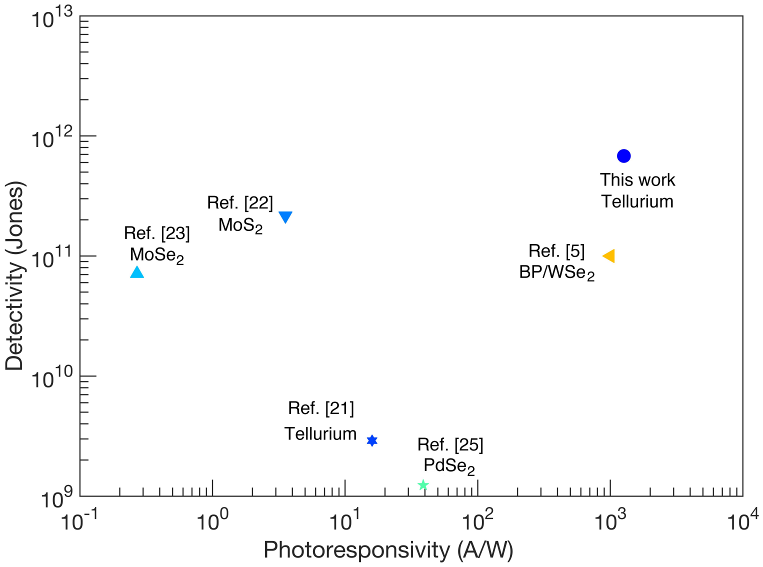

Supplementary Figure 26. Device performance comparison for various 2D materials.

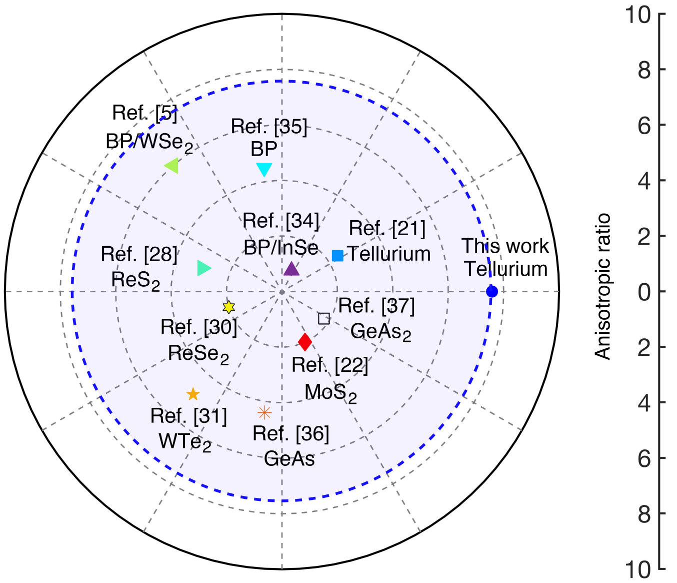

Supplementary Figure 27. Anisotropic ratio comparison of photoresponse for various 2D materials. The sensitive wavelength is limited from 0.52 μm to 2.3 μm.

Supplementary Table 1. Field effect mobility of different 2D materials

Material	Thickness	Mobility($cm^2V^{-1}s^{-1}$)	References
Tellurium	27.5 nm	938	This work
GeP	4.3 nm	200	10
Tellurium	16 nm	700	11
MoS_2	5 nm	28	12
ReS_2	28 layers	79.1	13
WS_2	3.5-5 nm	60	14
Black phosphorus	5 nm	286	15
Black phosphorus	10 nm	984	16
Bi_2O_2Se	5 nm	200	17
Tellurium	12.3 nm	419	21
$MoSe_2$	bulk	100	23
$PdSe_2$	14 nm	Electron:138.9<br>Hole: 57.0	25
ReS_2	3 nm	40	28
ReS_2	6 layers	15.4	29
$ReSe_2$	3 nm	10	30

Supplementary Table 2. Device performance comparison

Material	Wavelength range	Responsivity (R) (AW^{-1})	Detectivity (D_C^*) (Jones)	Response speed	Reference
Tellurium	Visible-MIR	1.36×10^3 @ 1.06 μm 3.54×10^2 @ 3.0 μm	7.69×10^{11} @ 1.06 μm 2.83×10^{11} @ 3.0 μm	Rise: 52.3 μs/ Fall: 64.0 μs @1.060 μm Rise: 56.0μs/ Fall: 66.0 μs @3.0 μm	This work
Black phosphorus/ WSe_2 heterostructure	Visible-NIR	1000 @ 0.637 μm 0.5 @ 1.55 μm	10^{10} @ NIR	Rise: 800 μs/ Fall: 800 μs @ 0.637 μm and 1.55 μm	5
WS_2/MoS_2 heterostructure	Visible	1090	3.5×10^{11}	Rise: 6.98 s/ Fall: 10.73 s	18
HgCdTe	Visible-LWIR	/	2×10^8	/	19
HgCdTe	Visible-LWIR	/	3.2×10^{10}	/	20
Tellurium	Visible-MIR	16	2.9×10^9	/	21
MoS_2	Visible-NIR	3.5	2×10^{11}	Rise: 0.4 ms/ Fall: 0.7 ms @ 0.52 μm and 0.83 μm	22
$MoSe_2$	Visible-NIR	0.27	7.13×10^{10}	Rise: 0.27 μs / Fall: 0.35 μs	23
$MoTe_2$	Visible-LWIR	4.15×10^{-5} @ 10.6 μm	10^8	At hundreds ms level	24
$PdSe_2$	Visible-LW	37.7	10^9	Rise: 51.3 μs/ Fall:	25

	IR	@ 3 μm		53.7 μs @ 0.637 μm<br>Rise: 74.5 ms/ Fall: 93.1 ms<br>@10.6 μm	
Black phosphorus	Visible-NIR	1000<br>@ 0.9 μm	/	Rise: 5 ms	26
Graphene/Ti_2O_3	Visible-LW IR	300	7×10^8	Rise: 1.2 ms/ Fall: 2.6 ms	27

Supplementary Table 3. Anisotropic ratio comparison

Material	Properties	Anisotropic ratio	Reference
Tellurium	Raman E_2 mode	12.5	This work
	Photoresponse	2.39 @ 1.55 μm<br>7.58 @ 2.3 μm	
Black phosphorus/WSe_2 heterostructure	Photoresponse	~6 @ 1.55 μm	5
Tellurium	Photoresponse	1.4 @ 1.55 μm<br>10 @ 3.0 μm	21
MoS_2	Photoresponse	2	22
ReS_2	Photoresponse	~3 @ 2.4 eV	28
ReS_2	Mobility	3	29
$ReSe_2$	Photoresponse	~2 @ 0.637 μm	30
WTe_2	Photoresponse	4.9	31
Black phosphorus	Thermal conductivity	2.1	32
Black phosphorus	Raman A_g^2 mode	~6	33
	Photoluminescence	~4.5	
Black phosphorus/InSe heterostructure	Photoresponse	0.83 @ 0.633 μm	34
Black phosphorus	Photoresponse	~4.5 @ 0.5 μm	35
GeAs	Photoresponse	1.49 @ 0.52 μm<br>4.4 @ 0.83 μm	36
$GeAs_2$	Conductance	~1.8	37
	Photoresponse	~1.8	
Black phosphorus-arsenic	Photoresponse	>100 @ 3.5 μm	38
	Absorption	~10 @ 3.5 μm	
Black phosphorus/ReS_2 heterostructure	Photoresponse	31	39
Black phosphorus	Photoresponse	3.5 @ 1.2 μm	40

	Reflectance	~2 (energies above the bandgap)	
	Transmittance	~2 @ 2.3 eV	

Supplementary References

1. Irmer, G., Röder, C., Himcinschi, C. & Kortus, J. Raman tensor elements and Faust-Henry coefficients of wurtzite-type α-GaN: How to overcome the dilemma of the sign of Faust-Henry coefficients in α-GaN? *J. Appl. Phys.* **116**, 245702 (2014).
2. Pine, A. S. & Dresselhaus, G. Raman spectra and lattice dynamics of tellurium. *Phys. Rev. B* **4**, 356-371 (1971).
3. Zhang, S. *et al.* Anomalous polarized Raman scattering and large circular intensity Differential in layered triclinic ReS_2. *ACS Nano* **11**, 10366-10372 (2017).
4. Wolverson, D., Crampin, S., Kazemi, A. S., Ilie, A. & Bending, S. J. Raman spectra of monolayer, few-layer, and bulk $ReSe_2$: an anisotropic layered semiconductor. *ACS Nano* **8**, 11154-11164 (2014).
5. Ye, L. *et al.* Highly polarization sensitive infrared photodetector based on black phosphorus-on-WSe_2 photogate vertical heterostructure. *Nano energy* **37**, 53-60 (2017).
6. Kwon, H. J., Kang, H., Jang, J., Kim, S. & Grigoropoulos, C. P. Analysis of flicker noise in two-dimensional multilayer MoS_2 transistors. *Appl. Phys. Lett.* **104**, 083110 (2014).
7. Tyo, J. S., Goldstein, D. L., Chenault, D. B. & Shaw, J. Review of passive imaging polarimetry for remote sensing applications. *Appl. Opt.* **45**, 5453-5469 (2006).
8. Myhre, G. *et al.* Liquid crystal polymer full-stokes division of focal plane polarimeter. *Opt. Express* **20**, 27393 (2012).
9. Gruev, V., Perkins, R. & York, T. CCD polarization imaging sensor with aluminum nanowire optical filters. *Opt. Express* **18**, 19087-19094 (2010).
10. Li, L. *et al.* 2D GeP: An unexploited low-symmetry semiconductor with strong in-plane anisotropy. *Adv. Mater.* **30**, 1706771 (2018).
11. Wang, Y. *et al.* Field-effect transistors made from solution-grown two-dimensional tellurene. *Nat. Electron.* **1**, 228-236 (2018).
12. Liu, H., Neal, A. T. & Ye, P. D. Channel length scaling of MoS_2 MOSFETs. *ACS Nano* **6**, 8563-8569 (2012).

13. Hämäläinen, J. *et al.* Atomic layer deposition of rhenium disulfide. *Adv. Mater.* **30**, 1703622 (2018).

14. Yang, L. *et al.* Chloride molecular doping technique on 2D materials: WS_2 and MoS_2. *Nano Lett.* **14**, 6275-6280 (2014).

15. Liu, H. *et al.* Phosphorene: an unexplored 2D semiconductor with a high hole mobility. *ACS Nano* **8**, 4033-4041 (2014).

16. Li, L. *et al.* Black phosphorus field-effect transistors. *Nat. Nanotechnol.* **9**, 372-377 (2014).

17. Zhang, Z. *et al.* Truly concomitant and independently expressed short- and long-term plasticity in a Bi_2O_2Se-based three-terminal memristor. *Adv. Mater.* 1805769, (2018).

18. Gong, F. *et al.* High-sensitivity floating-gate phototransistors based on WS_2 and MoS_2. *Adv. Funct. Mater.* **26**, 6084-6090 (2016).

19. Piotrowski, J.; Rogalski, A. Uncooled long wavelength infrared photon detectors. *Infra. Phys. Technol.* **46**, 115-131 (2004).

20. Rogalski, A. HgCdTe infrared detector material: history, status and outlook. *Rep. Prog. Phys.* **68**, 2267-2336 (2005).

21. Amani, M. *et al.* Solution-synthesized high-mobility tellurium nanoflakes for short-wave infrared photodetectors. *ACS Nano* **12**, 7253-7263 (2018).

22. Tong, L. *et al.* Artificial control of in-plane anisotropic photoelectricity in monolayer MoS_2. *Applied Mater. Today* **15**, 203-211 (2019).

23. Mao, J. *et al.* Ultrafast, broadband photodetector based on $MoSe_2$/Silicon heterojunction with vertically standing layered structure using graphene as transparent electrode. *Adv. Sci.* **3**, 1600018 (2016).

24. Lai, J. *et al.* Anisotropic broadband photoresponse of layered type-II Weyl semimetal $MoTe_2$. *Adv. Mater.* **30**, 1707152 (2018).

25. Long, M. *et al.* Palladium diselenide long-wavelength infrared photodetector with high sensitivity and stability. *ACS Nano* **13**, 2511-2519 (2019).

26. Huang, M. *et al.* Broadband black-phosphorus photodetectors with high responsivity. *Adv. Mater.* **28**, 3481-3485 (2016).

27. Yu, X., Narrow bandgap oxide nanoparticles coupled with graphene for high performance mid-infrared photodetection. *Nat. Commun.* **9**, 4299 (2018).

28. Liu, F. *et al.* Highly sensitive detection of polarized light using anisotropic 2D ReS_2. *Adv. Funct. Mater.* **26**, 1169-1177 (2016).

29. Liu, E. *et al.* Integrated digital inverters based on two-dimensional anisotropic ReS_2 field-effect transistors. *Nat. Commun.* **6**, 6991 (2015).

30. Zhang, E. *et al.* Tunable ambipolar polarization-sensitive photodetectors based on high-anisotropy $ReSe_2$ nanosheets. *ACS Nano* **10**, 8067-8077 (2016).

31. Zhou, W. *et al.* Anomalous and polarization-sensitive photoresponse of Td-WTe_2 from visible to infrared light. *Adv. Mater.* **31**, 1804629 (2018).

32. Luo, Z. *et al.* Anisotropic in-plane thermal conductivity observed in few-layer black phosphorus. *Nat. Commun.* **6**, 8572 (2015).

33. Wang, X. *et al.* Highly anisotropic and robust excitons in monolayer black phosphorus. *Nat. Nanotechnol.* **10**, 517-521 (2015).

34. Zhao, S. *et al.* Highly polarized and fast photoresponse of black phosphorus-InSe vertical p-n heterojunctions. *Adv. Funct. Mater.* **22**, 1802011 (2018).

35. Yuan, H. *et al.* Polarisation-sensitive broadband photodetector using black phosphorus vertical p-n junction. *Nat. Nanotechnol.* **10**, 707-713 (2015).

36. Zhou, Z. *et al*. Perpendicular optical reversal of the linear dichroism and polarized photodetection in 2D GeAs. *ACS Nano* **12**, 12416-12423 (2018).

37. Li, L. *et al*. Highly in‐plane anisotropic 2D $GeAs_2$ for polarization‐sensitive photodetection. *Adv. Mater.* **30**, 1804541 (2018).

38. Amani, M. *et al*. Mid-wave infrared photoconductors based on black phosphorus-arsenic alloys. *ACS Nano* **11**, 11724-11731 (2017).

39. Li, X. *et al*. Polarization-dependent photocurrent of black phosphorus/rhenium disulfide heterojunctions. *Adv. Mater. Interface*s **5**, 1800960 (2018).

40. Yuan, H. *et al*. Polarization-sensitive broadband photodetector using a black phosphorus vertical p-n junction. *Nat. Nanotechnol*. 10, 707-713 (2015).
